# Polyethylene Glycol Architecture Governs Interfacial Thermodynamics and Drug Adsorption in Anionic Lipid Microbubbles

**DOI:** 10.1021/acs.langmuir.6c03030

**Published:** 2026-09-12

**Authors:** L. Enrique Corona-Martínez, Amelia Ríos Rodríguez, J. Angel Navarro-Becerra

**Affiliations:** † 91087 Cinvestav−Monterrey, Apodaca, Nuevo León 66600, Mexico; ‡ FCFM−UANL, San Nicolás de Los Garza, Nuevo León 66451, Mexico; § SECIHTI, Mexico City 03940, Mexico

## Abstract

Polyethylene glycol (PEG)-stabilized lipid microbubbles (MBs) are promising ultrasound-responsive drug delivery platforms; however, their optimization remains largely empirical and fails to resolve the interfacial mechanisms governing drug adsorption and stability. Here, we established a thermodynamics-guided framework to determine how PEG grafting density, spanning mushroom to brush conformational regimes, regulates drug adsorption in anionic lipid MBs. Using doxorubicin (DOX) as a model drug, loading was quantified across macroscopic, mesoscopic, and microscopic descriptors, while adsorption energetics were evaluated through apparent Gibbs free energy (ΔG_app_), enthalpic (ΔH_app_) and entropic (ΔS_app_) contributions. Surface-normalized loading (DOX_molecules_/μm^2^) emerged as the most predictive descriptor of intrinsic apparent adsorption energetics, minimizing geometric and population-dependent variability across formulations. Increasing anionic lipid and PEG content progressively shifted adsorption from thermodynamically unfavorable to favorable regimes, accompanied by increasingly negative ΔG_app, interactions–SA_ values (from 4.6 ± 0.2 to −1.3 ± 0.9 kJ/mol). Mushroom architectures were dominated by hydrophobic interactions (56.0 ± 4.9%), intermediate regimes exhibited the strongest electrostatic contributions (77.1 ± 21.0%), whereas brush architectures promoted DOX–DOX π–π-dominated adsorption (78.9 ± 5.0%). Temperature-dependent studies revealed predominantly ΔH_app,SA_-driven adsorption, with brush architectures exhibiting the strongest thermodynamic stabilization (ΔH_app,SA_= −27.8 ± 4.0 kJ/mol). Correspondingly, brush MBs achieved up to 6.2 ± 1.5 × 10^6^ DOX_molecules_/μm^2^ without aggregation, representing ∼7.8-fold and ∼3.9-fold higher surface loading than mushroom and intermediate architectures, respectively. Together, these findings establish PEG architecture as a dominant regulator of adsorption energetics and loading behavior, providing predictive and rational design principles for engineering ultrasound-responsive MB drug delivery systems.

## Graphical abstract

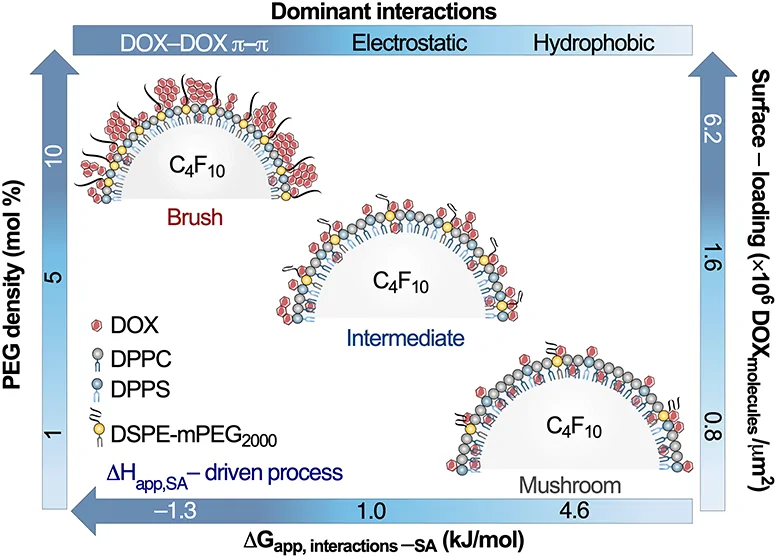

## 1 Introduction

Polyethylene glycol (PEG)-stabilized lipid microbubbles (MBs) are gas-filled microspheres, typically 1–10 μm in diameter, that have evolved from contrast-enhanced ultrasound (US) imaging agents into multifunctional platforms for molecular imaging and image-guided drug delivery.^1,2^ This transition has been driven by the incorporation of therapeutic payloads and targeting ligands, enabling controlled spatiotemporal release through acoustic cavitation.^1–3^ Current drug-loading strategies include covalent conjugation via PEG linkers,^4,5^ electrostatic and hydrophobic adsorption,^6,7^ and biotin–avidin coupling.^8,9^

Despite their widespread application, the characterization of drug-loaded MBs remains largely empirical, relying predominantly on macroscopic descriptors such as encapsulation efficiency and bulk yield.^1–3^ Although useful for initial formulation screening, these metrics reduce optimization to maximizing payload incorporation while overlooking the interfacial forces governing drug retention and stability. Consequently, formulation performance is frequently evaluated through storage stability^10,11^ and US-triggered release profiles,^11,12^ approaches that confirm functionality but remain fundamentally phenomenological. As a result, MB design remains constrained to a trial-and-error paradigm. Addressing this limitation requires frameworks capable of directly resolving interfacial loading energetics and the molecular interactions governing payload adsorption at the gas-liquid interface. Such mechanistic insight is essential to predict payload stability under dynamic physiological conditions and to enable the rational design of next-generation MB systems.

In pharmaceutical science and surface chemistry, thermodynamic analysis provides a rigorous framework for predictive, energy-based design and underpins the development of advanced drug delivery systems. Quantitative determination of the Gibbs free energy (ΔG), enthalpy (ΔH), and entropy (ΔS) enables mechanistic resolution of adsorption processes by distinguishing enthalpy-driven interactions from entropic contributions associated with desolvation and interfacial reorganization.^13,14^ Leveraging these principles enables the rational engineering design of drug carriers with enhanced efficacy, specificity, and safety.^15,16^ Hydrophobic and electrostatic interactions are particularly attractive for loading small molecules because they permit reversible association while preserving efficient US-triggered release.^2^ Within this context, doxorubicin (DOX), a widely used chemotherapeutic agent, serves as both a clinically relevant MB payload^12,17–19^ and a model probe for investigating MB interfacial behavior. Its cationic, amphiphilic structure promotes strong and reproducible interactions with charged lipid monolayers,^17,18,20^ while its adsorption is highly sensitive to variations in shell composition and interfacial free energy. This sensitivity provides a powerful platform for quantifying the molecular-scale interactions governing payload adsorption and interfacial stability.

To balance DOX loading capacity with therapeutic performance, MB formulations typically combine zwitterionic phospholipids for structural integrity, anionic lipids for electrostatic regulation, and PEGylated lipids for steric stabilization.^11,12,17–19^ Although the influence of anionic lipid composition on DOX loading^17,18^ and acoustic response,^12,18^ is well-established, the role of PEG architecture remains poorly understood. PEG is not merely a passive stabilizer but a dominant regulator of interfacial structure and mechanics. As PEG grafting density and molecular weight increase, tethered chains undergo a conformational transition from sparsely distributed mushroom regimes to densely packed brush layers.^21–23^ This transition modulates shell elasticity, suppresses coalescence, and alters MB acoustic behavior.^21,24–27^

Although PEGylation is conventionally employed to prolong circulation time by reducing protein adsorption,^28,29^ recent studies demonstrates that PEG can paradoxically enhance immunogenicity and accelerate MB clearance.^30^ We recently showed that PEG architecture actively reprograms MB pharmacokinetics, where mushroom-like coatings prolong circulation while dense brush layers promote rapid clearance via complement activation.^22^ Increasing PEG surface densities further induces thermodynamically driven lateral phase separation within lipid monolayers,^31^ generating interfacial domains that may spatially regulate drug adsorption. Collectively, these findings identify PEG architecture as a key regulator of interfacial thermodynamics, adsorption pathways, and drug-loading behavior.

Here, we aimed to establish a thermodynamics-guided framework to elucidate how PEG organization governs drug adsorption and loading in anionic lipid MBs. By systematically varying PEG grafting density across mushroom, intermediate and brush conformational regimes, we quantified the Gibbs free energy and its enthalpic and entropic contributions governing DOX adsorption across macroscopic, mesoscopic, and microscopic interfacial length scales. We further evaluated how drug concentration and incubation time modulate adsorption within distinct PEG architectures, establishing a direct mechanistic relationship between shell organization, adsorption energetics, and tunable loading performance.

## 2 Materials and Methods

### 2.1 Materials

Dipalmitoyl-*sn*-glycero-3-phosphocholine (DPPC), 1,2-dipalmitoyl-*sn*-glycero-3-phospho-l-serine (sodium salt; DPPS), dextrose, chloroform, glycerol, and propylene glycol were purchased from Sigma-Aldrich (St. Louis, MO, USA). The PEGylated lipid 1,2-distearoyl-*sn*-glycero-3-phosphoethanolamine-N-[methoxy­(polyethylene glycol)-2000] (DSPE-mPEG_2000_) was acquired from NOF America Corporation (White Plains, NY, USA). Perfluorobutane gas (C_4_F_10_, PFB) was obtained from FluoroMed (Round Rock, TX, USA). Medical-grade doxorubicin hydrochloride (ZUCLODOX^MR^, DOX) was obtained from Zurich Pharma S.A. de C.V. (Tepeji del Río, Hidalgo,México). All reagents were used as received and possessed a purity ≥99%.

### 2.2 Theoretical Classification of PEG Shell Architectures

MB shell architectures were systematically engineered to generate mushroom, intermediate, and brush PEG conformational regimes by varying the molar fraction of DSPE-mPEG_2000_ (χ_PEG_), as previously described.^22^ PEG conformations were assigned according to the classical Alexander–de Gennes polymer brush theory,^23^ in which chain organization is governed by the balance between the Flory radius (R_F_) and the average inter-chain distance (D). R_F_ represents the characteristic size of an isolated PEG chain in a good solvent, whereas D corresponds to the mean spacing between neighboring grafting sites and is related to the PEG grafting density (σ), defined as the number of grafted PEG chains per unit of surface area. Assuming a homogeneous PEG-lipid distribution, σ is given by σ = χ_PEG_/A_lipid_,^21,23^ yielding:

1 $$D={\left(\frac{1}{\sigma }\right)}^{1/2}=\sqrt{\frac{A_{\mathrm{lipid}}}{X_{\mathrm{PEG}}}}$$

PEG conformational regimes were determined by the relative magnitude of D and R_F_, with mushroom (D > R_F_), intermediate (D ≈ R_F_), and brush (D < R_F_) regimes.^21–23^ Accordingly, χ_PEG_ values of 0.01, 0.05, and 0.10 correspond to mushroom, intermediate, and brush conformational regimes, respectively (Table 1).

**Table 1:** Theoretical PEG Configuration Parameters for Engineered MB Shells

χ_PEG_	D (nm)	L (nm)	Architecture
0.01	6.7	2.2	Mushroom
0.05	3.0	3.8	Intermediate
0.10	2.1	4.7	Brush

Structural parameters were calculated as follows:

2 $$R_{F}{=\mathrm{aN}}^{3/5}$$

3 $$D=\sqrt{\frac{A_{\mathrm{lipid}}}{X_{\mathrm{PEG}}}}$$

4 $$L=\mathrm{aN}(\frac{a}{D}{)}^{2/3}$$

where a is the PEG monomer length (0.35 nm), N is the number of monomers per chain (45 for PEG_2000_).^21^ A_lipid_ is the average cross-sectional area of the lipid headgroups in the gel phase (0.452 nm^2^), obtained from reported values for DPPC (0.473 nm^2^), DPPS (0.442 nm^2^) and DSPE (0.440 nm^2^).^32–34^ L denotes the mean height of grafted PEG chains above the interface. Calculated values of R_F_, D, and L are summarized in Table 1.

### 2.3 Preparation of Anionic PEGylated MBs

Anionic PEGylated C_4_F_10_ gas-core MBs were prepared by mechanical agitation as previously described.^6,35^ Lipid shells consisted of DPPC, DPPS and DSPE-mPEG_2000_. Surface charge and polymer architecture were independently tuned by varying the molar fractions of DPPS (χ_DPPS_) and PEG (χ_PEG_), respectively, as summarized in Table 2.

**Table 2:** Lipid Shell Compositions of Engineered MBs

χ_PEG_	χ_DPPS_				χ_DPPC_			
0.01	0.00	0.10	0.20	0.40	0.99	0.89	0.79	0.59
0.05	0.00	0.20	0.30	0.40	0.95	0.75	0.65	0.55
0.10	0.00	0.20	0.30	0.40	0.90	0.70	0.60	0.50

Briefly, individual lipids components were dissolved in their respective organic solvents: DPPC and DSPE-mPEG_2000_ in chloroform and DPPS in chloroform/methanol (2:1, v/v). Then, the lipids were combined at the desired molar ratios (Table 2), and the solvent was evaporated under a gentle nitrogen stream. Residual solvent was eliminated under vacuum for 2 h to obtain dry lipid films. The resulting lipid films were hydrated in phosphate-buffered saline (PBS, pH 7.4) containing glycerol and propylene glycol (80:10:10 v/v) at 60 °C for 40 min to achieve a final total lipid concentration of 2.0 mg/mL. Subsequently, 0.5 mL of the liposomal suspension was transferred to a 1.5 mL glass vials, sealed, degassed and refilled with C_4_F_10_ gas for 10 s. Polydisperse MB suspensions were generated using an Ultramat S dental amalgamator (SDI, Effingham, IL, USA) operated at 4500 osc/s for 46 s. Immediately after activation, the MB suspension was transferred to sealed 0.65 mL microcentrifuge tubes for purification. Excess lipids and emulsifiers were removed by a single centrifugation step (300 × g, 10 min). Following centrifugation, the lower aqueous phase (subnatant) was carefully removed, and the floating MB cake was collected and stored at 4 °C no longer than 1 h prior to further use.

### 2.4 Preparation of DOX-Loaded MBs

DOX-loaded MBs (DOX–MBs) were prepared by incubating preformed anionic PEGylated MBs with freshly reconstituted DOX solution in non-degassed sterile water (2 mg/mL, pH = 8.2). The DOX loading procedure was adapted from a previously reported formulations.^17,19^ Prior to loading, the DOX stock solution was diluted with sterile water containing 5 mg/mL dextrose to obtain a final DOX concentration of 0.5 mg/mL. The collected MB cake of each PEGylated MB formulation was then resuspended in 150 μL of the diluted DOX solution and incubated under gentle rotational agitation for 10 min. The following experimental protocols were designed to resolve the thermodynamic mechanisms governing DOX adsorption:

1) Baseline adsorption and apparent Gibbs free energy (ΔG_app_): Baseline adsorption was defined as the amount of DOX adsorbed onto each anionic PEGylated formulation (Table 1) under the reference condition of 25 °C (298.15 K). These values were quantified from the DOX loading measurements obtained under the standard loading condition (0.5 mg/mL DOX and 10 min incubation) using the loading descriptors described in Section 2.6. The resulting baseline adsorption values were used as reference data for the calculation of ΔG_app_, as described in Section 2.7.

2) Apparent enthalpic (ΔH_app_) and entropic (ΔS_app_) contributions: To evaluate the temperature dependence of adsorption, the baseline adsorption protocol was independently performed at 4 °C (277.15 K) and 37 °C (310.15 K), representing relevant storage and physiological conditions, respectively.

Following incubation, DOX–MB suspensions were washed three times by centrifugation (300 × g, 5 min) to remove unbound or weakly associated DOX remaining in the surrounding medium. Three washing cycles were applied until the absorbance values of the supernatant reached levels comparable to sterile water, minimizing the contribution of residual free DOX to the colorimetric measurement. Samples were subsequently resuspended in 150 μL of non-degassed sterile water and transferred to 0.2 mL microcentrifuge tubes under ambient air headspace. Samples were stored at 4 °C for no longer than 1 h prior to microscopic and spectrophotometric characterization to minimize potential changes in DOX–MB properties.

### 2.5 Characterization of DOX–MBs

DOX–MB suspensions were diluted 1:100 in buffer and loaded into a Neubauer counting chamber (Hausser Scientific, Horsham, PA, USA). Following our previously reported methodology,^22,36^ for each independent batch, four microscopy images were acquired from each of the four Neubauer quadrants using an Olympus BX51TF microscope equipped with a 50× objective and a Photron FastCAM SA3 camera (Tokyo, Japan). Each image was acquired at a resolution of 768 × 768 pixels and encompassed a field of view equivalent to a single counting square within the Neubauer quadrant. A total of 16 images were collected, corresponding to the standard counting area of one complete Neubauer quadrant. Image analysis was performed using ImageJ software (National Institutes of Health, Bethesda, MD, USA). Images were processed using a kernel convolution and convolution filters to identify MB structures. After binarization, particles were sized and quantified using the *Analyze particles script*, applying a circularity threshold of >0.5 for individual MBs and <0.5 for agglomerates.

MB concentration (C_MB_) was determined by microscopic analysis using the Neubauer chamber rule ( $C_{MB}=(MBs_{counted}\times dilution\;factor\times \frac{10^{4}}{mL})$ .^36^ To evaluate DOX adsorption independently of variations in the initial C_MB_, raw number-weighted size distributions were normalized to a reference concentration (C_ref_ = 1 × 10^10^ MBs/mL), as previously proposed to facilitate direct comparisons among MB formulations.^1,36^ The mean-weighted diameter (D_m_) and the standard deviation (SD) were calculated from the normalized particle number distributions.

To determine the SA distributions, individual MB diameters (*d*_*i*_) were converted to surface areas $\left(s=\pi d_{i}^{2}\right)$ . The normalized number-weighted size distribution was then transformed into a SA distribution by calculating SA contribution of each interval as *SA*(*s*) = *N*(*s*) × *s*, where N (s) is the normalized particle number corresponding to a given MB surface area (MB_SA_). The cumulative interfacial SA available for adsorption (SA_normalized_) was calculated as the area under the SA distribution:

5 $${\mathrm{SA}}_{\mathrm{normalized}}=\int_{S_{\min}}^{S_{\max}}SA\left(s\right)\mathrm{ds}$$

### 2.6 Determination of Encapsulated Drug and Loading Descriptors

Encapsulated DOX was quantified using an adapted version of our previously reported method.^4–6^ A DOX standard curve (0.0–1.0 mg/mL) was prepared in sterile water prior to analysis. DOX–MBs were disrupted by US exposure for 60 s using an ultrasonic bath (Fisher Scientific FS30, Pittsburgh, PA, USA) to release encapsulated drug. Samples were transferred into 96-well plates (Costar, Corning, NY, USA) and absorbance was measured at 496 nm using a microplate reader (BioTek Instruments ELx800, Winooski, VT, USA). Sterile water served as the blank control, and disrupted unloaded MBs were used to assess background absorbance from MB lipid components. Encapsulation efficiency (EE%) was calculated as

$$\mathrm{EE}\%=\frac{{\mathrm{DOX}}_{\mathrm{detected}}}{{\mathrm{DOX}}_{\mathrm{added}\left(\mathrm{mg}\right)}}\times 100\%$$ 6

To establish a multi-scale adsorption framework, drug loading was quantified at macroscopic (encapsulated mass, DOX_mass_), mesoscopic (molecules per MB, DOX_molecules_/MB), and microscopic (surface density, DOX_molecules_/μm^2^) scales. All descriptors were normalized to a C_ref_ of 1 × 10^10^ MBs/mL. The descriptors were calculated as follows:

7 $${\mathrm{DOX}}_{\mathrm{mass}}=\frac{M_{\mathrm{DOX}/\mathrm{mL}}}{C_{\mathrm{MB}}}\;{\times C}_{\mathrm{ref}}$$

8 $${\mathrm{DOX}}_{\mathrm{molecules}/\mathrm{MB}}=\frac{{\mathrm{DOX}}_{\mathrm{mass}\;}{\times N}_{A}}{{\mathrm{MW}}_{\mathrm{DOX}\;}{\times C}_{\mathrm{ref}}}$$

9 $${\mathrm{DOX}}_{{\mathrm{molecules}/\mu m}^{2}}=\frac{({\mathrm{DOX}}_{\mathrm{mass}}{/\mathrm{MW}}_{\mathrm{DOX}})\;{\times N}_{A}}{{\mathrm{SA}}_{\mathrm{total}}}$$

where M_DOX/mL_ is the detected mass of DOX per mL, C_MB_ is the measured MBs concentration, N_A_ is Avogadro’s number (N_A_ = 6.022 × 10^23^ molecules/mol), MW_DOX_ is the molecular weight of DOX (579.98 g/mol), and S*A*_total_ is the cumulative SA per mL of the standardized MB population.

### 2.7 Thermodynamic Analysis of Drug Adsorption

#### 2.7.1 Adsorption Modeling

DOX adsorption data associated with each loading descriptor were analyzed using simplified phenomenological adsorption models to characterize the apparent dependence of interfacial uptake on bulk DOX concentration and lipid shell composition. At low interfacial coverage, adsorption was described using a Henry-type adsorption regime: q_e_ = K_H_C_e_.^37^ Where q_e_ is the interfacial concentration, K_H_ is the Henry-type adsorption constant, and C_e_ is the DOX bulk concentration. To evaluate the influence of lipid shell composition on adsorption, the dependence of DOX uptake on χ_DPPS_ was described using the linear relationship:

$$q_{e}=k\chi_{\mathrm{DPPS}}+b$$ 10

where *k* is the sensitivity coefficient describing the dependence of adsorption on χ_DPPS_, and b represents baseline adsorption at χ_DPPS_ = 0.0, corresponding to adsorption on DPPC/DSPE-mPEG_2000_ shells in the absence of DPPS.

The nonlinear increase in DOX adsorption was analyzed using a simplified Gouy–Chapman–Boltzmann framework relating interfacial concentration (Γ, DOX encapsulated) to surface potential (ψ_0_): ${\Gamma =\Gamma }_{0}e^{\left(-\frac{\mathrm{zF}\psi_{0}}{\mathrm{RT}}\right)}$ .^38^ Where Γ_0_ is the DOX encapsulated in absence of DPPS, *z* is the valency of DOX (z = +1), F is the Faraday constant (96,485 C/mol), R is the ideal gas constant (8.314 J·mol^‑1^·K^‑1^), and T is the absolute temperature (K).

Assuming that ψ_0_ scales linearly with χ_DPPS_ at low potentials (ψ_0_ – 25 mV), the model simplifies to

$${\Gamma =\Gamma }_{0}e^{k\;\chi_{\mathrm{DPPS}}}$$ 11

Experimental data were fitted to both linear and nonlinear regimes. The preferred model was selected based on the highest R^2^ value, and the corresponding *k* value was used to quantify the dependence of DOX adsorption on χ_DPPS_.

#### 2.7.2 Apparent Gibbs Free Energy of Adsorption

The apparent Gibbs free energy of adsorption (ΔG_app_) was determined as a comparative descriptor of the apparent adsorption affinity of DOX toward the MB shell. ΔG_app_ was calculated from the dimensionless apparent adsorption coefficient (K_app_),^38^ obtained from the loading descriptors described in Section 2.6:

12 $${\Delta G}_{\mathrm{app}}=-\mathrm{RTln}\left(K_{\mathrm{app}}\right)$$

where $K_{\mathrm{app}}=\frac{DOX_{encapsulated}}{DOX_{added}}$ , DOX_encapsulated_ is the experimentally measured amount of DOX associated with the MB shell, and DOX_added_ is the total amount of DOX introduced during the loading. Because K_app_ is derived from loading descriptors rather than from a thermodynamic adsorption equilibrium constant, ΔG_app_ should be interpreted as an apparent adsorption free-energy descriptor (ΔG_app, mass_, ΔG_app, MB_ *or* ΔG_app, SA_).

Based on previous studies describing DOX adsorption at anionic phospholipid interfaces, the intermolecular interactions considered in this framework included hydrophobic interactions with lipid hydrocarbon domains, electrostatic interactions with anionic lipid components (DPPS and DSPE-mPEG_2000_)^20,39^ and DOX–DOX π–π self-association.^40^ The relative contributions of electrostatic, hydrophobic, and DOX–DOX π–π interactions were estimated using a hierarchical interaction framework based on the interaction range. Electrostatic interactions were assigned first because their longer interaction range is expected to govern the initial association of DOX with anionic DPPS and DSPE-mPEG_2000_ headgroups.^41^ Hydrophobic interactions were subsequently assigned as short-range lipid-associated interactions,^42^ followed by DOX–DOX π–π stacking, which requires close proximity between aromatic rings.^43^

The electrostatic contribution was estimated as

13 $$\%\;\mathrm{Electrostatic}=\frac{N_{\mathrm{DPPS}\;}{+N}_{{\mathrm{DSPE}‐\mathrm{mPEG}}_{2000}}}{N_{DOX}}\times 100\%$$

where N_DPPS_ and $N_{DSPE‐mPEG_{2000}}$ correspond to the number of available anionic lipid molecules and N_DOX_ is the total number of adsorbed DOX molecules.

At χ_DPPS_ = 0.0, adsorbed DOX molecules exceeding the electrostatic capacity of DSPE-mPEG_2000_ were attributed to hydrophobic interactions:

$$\%\;\mathrm{Hydrophobic}=\frac{N_{hydrophobic_{\left(\chi_{DPPS\;=\;0.0}\right)}}}{N_{DOX}}\times 100\%$$ 14

where $N_{hydrophobic_{(\chi_{DPPS\;=\;0.0)}}}=N_{DOX}-N_{{\mathrm{DSPE‐mPEG}}_{2000}}$ if $N_{DOX}>N_{hydrophobic_{\left(\chi_{DPPS=0.0}\right)}}+(N_{DPPS}+N_{{\mathrm{DSPE‐mPEG}}_{2000})}$ the remaining adsorbed DOX molecules were assigned to DOX–DOX π–π interactions:

% π−π=100%−(% Hydrophobic+% Electrostatic) 15

The number of lipid molecules (N_lipid_) for each lipid species (DPPC, DPPS, or DSPE-mPEG_2000_) per 1 × 10^10^ MBs was calculated as $N_{lipid}=\frac{\chi_{\mathrm{lipid}\;}\times \;MB_{SA,\;total}}{A_{lipid}}$ , where χ*_lipid_* is the molar fraction of the corresponding lipid, *MB_SA, total_* is the total MB surface area normalized to 1 × 10^10^ MBs, and A_lipid_ is the molecular area per lipid.

Finally, following classical approaches for describing interfacial free-energy contributions,^44^ the ΔG_app,SA_ was interpreted as the combined contribution of these interaction regimes:

16 $${\Delta G}_{\mathrm{app},\;\mathrm{interactions}\;}{=\Delta G}_{\mathrm{app},\;\mathrm{hydrophobic}\;}{+\Delta G}_{\mathrm{app},\;\mathrm{electrostatic}\;+\mathrm{DOX}-\mathrm{DOX}(\pi ‐\pi )}$$

where ${\Delta G}_{\mathrm{app},\;\mathrm{hydrophobic}}=-\mathrm{RT}\ln\left(K_{app,\;hydrophobic}\right)$ , and ${\Delta G}_{\mathrm{app},\;\mathrm{electrostatic}\;+\mathrm{DOX}-\mathrm{DOX}(\pi ‐\pi )}=-RT\ln\left(K_{app,\;electrostatic\;+\mathrm{DOX}-\mathrm{DOX}(\pi ‐\pi )}\right)$ , where $K_{app,\;hydrophobic}=\frac{N_{hydophobic_{(\chi_{DPPS\;=\;0.0)}}}}{N_{DOX}}$ , $K_{app,\;electrostatic\;+\mathrm{DOX}-\mathrm{DOX}(\pi ‐\pi )}=e^{k(\chi_{DPPS\;\;}+\chi_{DSPE‐{mPEG}_{2000}})}$ and *k* is the sensitivity coefficient obtained from the fits of the linear and nonlinear adsorption models described in Section 2.7.1 for each multiscale loading descriptor (Section 2.6).

#### 2.7.3 Apparent Enthalpic and Entropic Contributions

To resolve the thermodynamic drivers of adsorption, temperature-dependent adsorption experiments were performed at 277.15, 298.15, and 310.15 K. The baseline adsorption protocol described above (Section 2.5) was independently conducted at each temperature. The temperature-dependent thermodynamic analysis was performed using the microscopic descriptor (DOX_molecules_/μm^2^).

The temperature dependence of ΔG_app, interactions–*SA*_ was described according to^38^

17 $${\Delta G}_{\mathrm{app},\;\mathrm{interactions}-SA}={\Delta H}_{app,\;SA}-T{\Delta S}_{app,\;SA}$$

Apparent enthalpic (ΔH_app, SA_) and entropic (ΔS_app, SA_) contributions were determined from the linearized van’t Hoff equation:^45^

18 $${\ln(K}_{\mathrm{app},\;\mathrm{interactions}-\mathrm{SA}})=\left(-\frac{{\Delta H}_{app,\;SA}}{R}\right)\frac{1}{T}+\frac{{\Delta S}_{app,\;SA}}{R}$$

where $K_{app,\;interactio\mathrm{ns}-SA}=K_{app,\;hydrophobic}+K_{app,\;electrostatic+\mathrm{DOX}-\mathrm{DOX}(\pi ‐\pi )}$ corresponds to the apparent adsorption coefficient calculated from the experimentally calculated SA loading descriptor at each temperature, as described in Section 2.7.2. Accordingly, ΔH_app,SA_ and ΔS_app,SA_ were obtained from the slope $\left(m=-\frac{{\Delta H}_{app}}{R}\right)$ and intercept $\left(b=\frac{{\Delta S}_{app}}{R}\right)$ of the linear regression of ln *K*_app, interactions–SA_ *versus* 1/T, respectively.

### 2.8 Analysis of Drug Loading Capacity

To determine the maximum DOX loading capacity while minimizing agglomeration, MBs exhibiting intermediate and brush PEG configurations were incubated at 298.15 K using DOX concentrations ranging from 0.5 to 2.0 mg/mL and incubation times from 10 to 60 min. Following incubation, samples were processed and characterized as described above.

### 2.9 Statistical Analysis

Each experimental group consisted of five independent anionic PEGylated-MB batches prepared on different days (n = 5). Data are presented as the mean ± standard deviation (SD). Statistical significance between experimental groups was evaluated using a one-way analysis of variance (ANOVA) followed by Tukey’s post-hoc test for multiple comparisons, whereas pairwise comparisons were assessed using an unpaired t-tests. Statistical analyses were generated using GraphPad Prism software (San Diego, CA, USA). A p value of p ≤ 0.05 was considered statistically significant.

## 3 Results and Discussion

### 3.1 PEG-DOX Microbubble Characterization

Anionic PEGylated MBs with controlled PEG architectures—mushroom (1 mol %), intermediate (5 mol %), and brush (10 mol %)—were successfully loaded with DOX (0.5 mg/mL of DOX, 10 min incubation) yielding predominantly spherical and well-dispersed populations under most conditions (Figure 1A). The corresponding lipid compositions (χ_DPPC_, χ_DPPS_ and χ_PEG_) for each PEG architecture are summarized in Table 2. Additional microscopy fields corresponding to one complete quadrant of the Neubauer chamber are provided in Figure S1 to illustrate the MB populations used for quantitative analysis. DOX–MBs at χ_DPPS_ = 0.1 and 0.2 were morphologically similar to those at χ_DPPS_ = 0.0.

**Figure 1:**
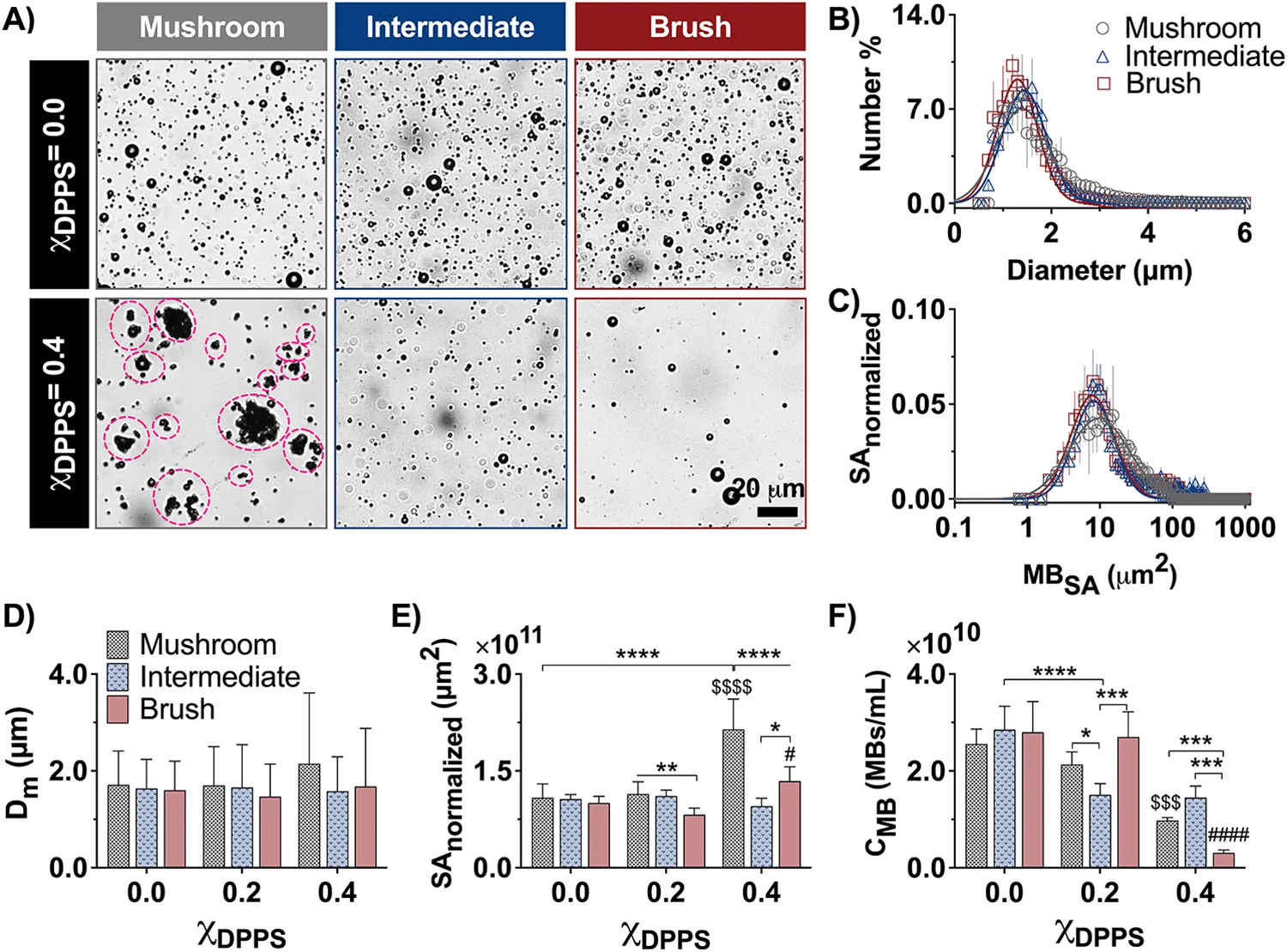
Characterization of DOX–MBs as a function of PEG architecture and anionic lipid content. A) Representative brightfield micrographs of mushroom, intermediate and brush MBs at low (χ_DPPS_ = 0.0) and high (χ_DPPS_ = 0.4) anionic lipid content after DOX loading (0.5 mg/mL, 10 min). Fuchsia dashed outlines indicate MB aggregation. B) Number-weighted size distributions and C) corresponding surface area distributions for χ_DPPS_ = 0.2, normalized to 1 × 10^10^ MBs/mL. Solid lines represent Gaussian fits (R^2^ ≥ 0.95). (D–F) Number-weighted mean diameter (D_m_), normalized SA (SA_normalized_), and MB concentration yield (C_MB_). Data represent the mean ± standard deviation of five independent experiments. Statistical significance is indicated as ^*, **, ***, ****^p < 0.05; ^$$$,$$$$^p < 0.005 *vs.* mushroom at χ_DPPS_ = 0.2 and ^#, ####^p < 0.05 *vs.* brush at χ_DPPS_ = 0.2.

At high anionic content (χ_DPPS_ = 0.4), mushroom MBs exhibited reduced colloidal stability and formed large aggregates (3–20 μm range; Figure 1A, and S1). The aggregate concentration (C_Agg_ = 1.5 ± 0.6 × 10^10^, mean diameter (D_m_= 10.2 ± 4.3 μm), and normalized SA (SA_normalized_ = 1.4 ± 0.6 × 10^12^ μm^2^) are shown in Figure S2. This behavior is consistent with enhanced DOX adsorption through hydrophobic and electrostatic interactions,^12,17,18^ which have been reported to promote DOX–DOX π–π stacking between anthracycline moieties^46^ and cooperative inter-MB aggregation.^17,18^ Although stable DOX–MBs have been reported at comparable PEG densities,^12,18^ those formulations incorporated DOX during lipid assembly. In contrast, adsorption onto preformed MBs leaves DOX exposed at the lipid interface, where the low PEG grafting density provides limited steric shielding. This reduced steric protection is associated with the mushroom PEG configuration, which exhibits larger D and shorter L compared with intermediate and brush architectures (Table 1), providing lower steric–diffusional hindrance. Under high χ_DPPS_ conditions, enhanced electrostatic DOX–lipid interactions and limited PEG shielding promote DOX–DOX association between neighboring MBs, leading to cooperative inter-MB aggregation within a short incubation period (Figure 2A). By contrast, intermediate and brush architectures remained colloidally stable under these identical conditions (10 min, 0.5 mg/mL DOX), consistent with increased PEG grafting density providing steric–diffusional hindrance^29,47,48^ that limits interfacial DOX–DOX interactions and suppresses DOX–mediated MB aggregation.

**Figure 2:**
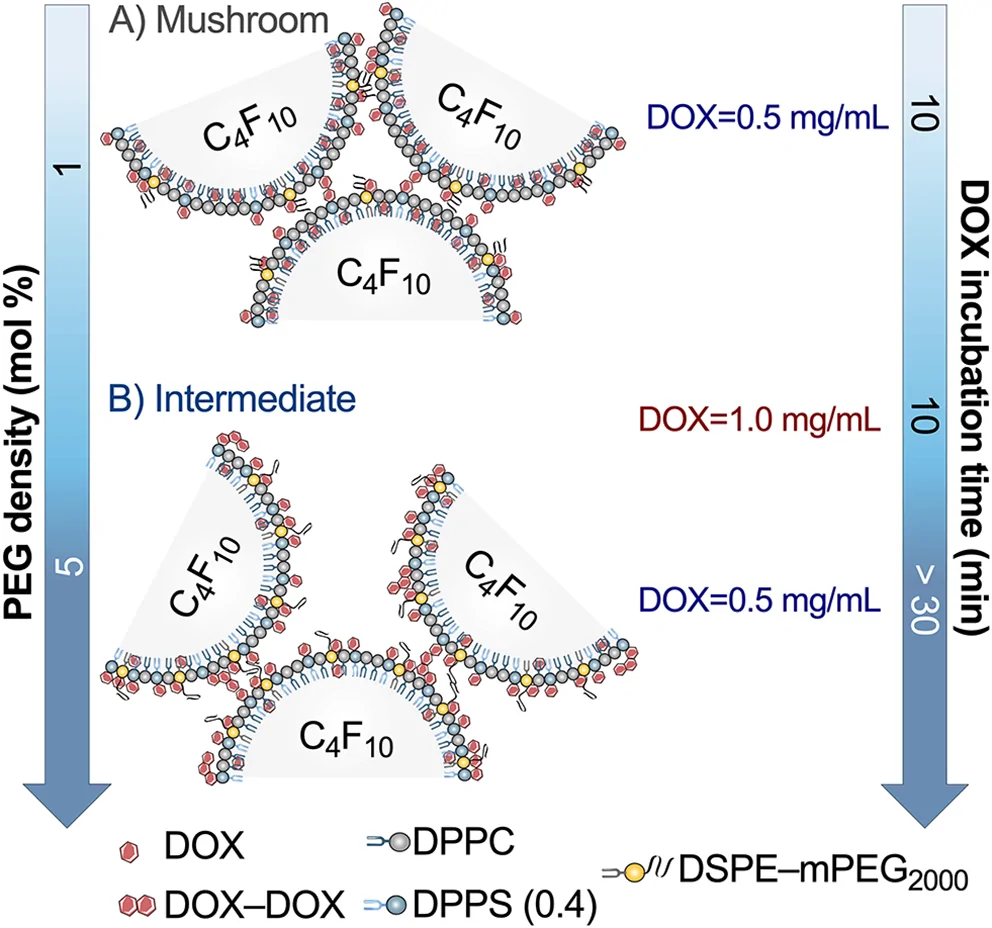
Schematic illustration of the proposed mechanism underlying PEG architecture-dependent, DOX-mediated cooperative inter-MB aggregation through intermolecular DOX–DOX interactions at the MB interface. (A) Mushroom and (B) intermediate PEG-coated MBs at χ_DPPS_ = 0.4 showing the DOX concentration- and incubation time-dependent aggregation behavior.

All non-aggregated formulations exhibited broad but well-defined size distributions (0.7–6.0 μm; Figure 1B and Figure S3) well described by Gaussian fits (R^2^ ≥ 0.90). Mean diameter remained nearly invariant across χ_DPPS_ (D_m_ = 1.40 ± 0.50 μm, Figure 1D), consistent with previous PEGylated MB formulations.^21,22,25,26^ At χ_DPPS_ = 0.4, aggregation dominated mushroom MBs, whereas brush MBs exhibited a shift toward larger individual MBs (6–10 μm, Figure S3B) without evidence of aggregation. Likewise, SA_normalized_ per 1.0 × 10^10^ MBs/mL remained ∼1.1 ± 0.5 × 10^11^μm^2^ across most formulations (Figure 1C). Normalization to a fixed MB concentration enabled direct comparison of size-dependent surface area distributions despite formulation-dependent differences in MB concentration. An increase was observed only at χ_DPPS_ = 0.4 for mushroom and brush MBs (2.2 ± 0.5 × 10^11^ and 1.3 ± 0.2 × 10^11^ μm^2^, respectively; Figure 1E).

MB concentration decreased monotonically with increasing χ_DPPS_ (Figure 1F), reaching minimum values of 9.7 ± 0.6 × 10^9^, 1.4 ± 0.2 × 10^10^, and 6.4 ± 0.3 × 10^9^ MBs/mL for mushroom, intermediate,

brush MBs, respectively. Similar MB concentrations were obtained before DOX incubation, indicating that this trend arises from increasing DPPS content rather than the adsorption process. Negatively charged phospholipids are known to alter lipid packing and interfacial organization through electrostatic repulsion between lipid headgroups,^49^ which likely reduces MB formation efficiency as χ_DPPS_ increases. Nevertheless, all formulations remained within clinically relevant concentration ranges for ultrasound contrast agents (5 × 10^8^–1.2 × 10^10^ MBs/mL),^1^ supporting translational feasibility. Collectively, these results highlight the role of PEG architecture and anionic lipid composition in determining the colloidal stability and morphological properties of DOX–MBs.

### 3.2 Multiscale Analysis of DOX Encapsulation

DOX encapsulation was initially quantified using encapsulation efficiency (EE%) as a bulk descriptor of loading yield (Figure 3A). Across all PEG architectures, EE% increased monotonically with χ_DPPS_, consistent with enhanced electrostatic interactions between DOX and the anionic lipid monolayer.^20^ Conversely, EE% decreased with increasing PEG density, indicating that densely packed PEG layers impose a steric constraints that limit bulk DOX loading.^48,50^ Mushroom MBs exhibited the highest EE% at low and intermediate χ_DPPS_ (22.0 ± 1.1% and 33.0 ± 2.0%, respectively), whereas χ_DPPS_= 0.4 was excluded due to extensive aggregation (Figure 1A and Figure S1). Intermediate MBs showed the largest relative increase in EE% (13.0 ± 2.2% to 38.0 ± 5.0%), whereas brush MBs reached 18.0 ± 4.0% at χ_DPPS_= 0.4. These trends are consistent with previous studies employing low PEG densities to maximize DOX loading and acoustic response.^12,18^ However, although higher PEG densities have demonstrated therapeutic efficacy,^11,17,19^ their effects on interfacial adsorption and molecular loading remain poorly understood. Importantly, EE% is a bulk descriptor of loading yield and does not directly account for formulation-dependent variations in MB concentration, size distribution, and accessible interfacial area. Although the D_m_ remained relatively constant across formulations (Figure 1D), differences in MB concentration (Figure 1F) resulted in distinct total surface area available for DOX adsorption, preventing direct comparison of size-dependent surface area contributions unless size distributions are normalized to a common MB concentration (Figure 1E). Therefore, EE% alone provides limited mechanistic insight into architecture-dependent DOX loading.

**Figure 3:**
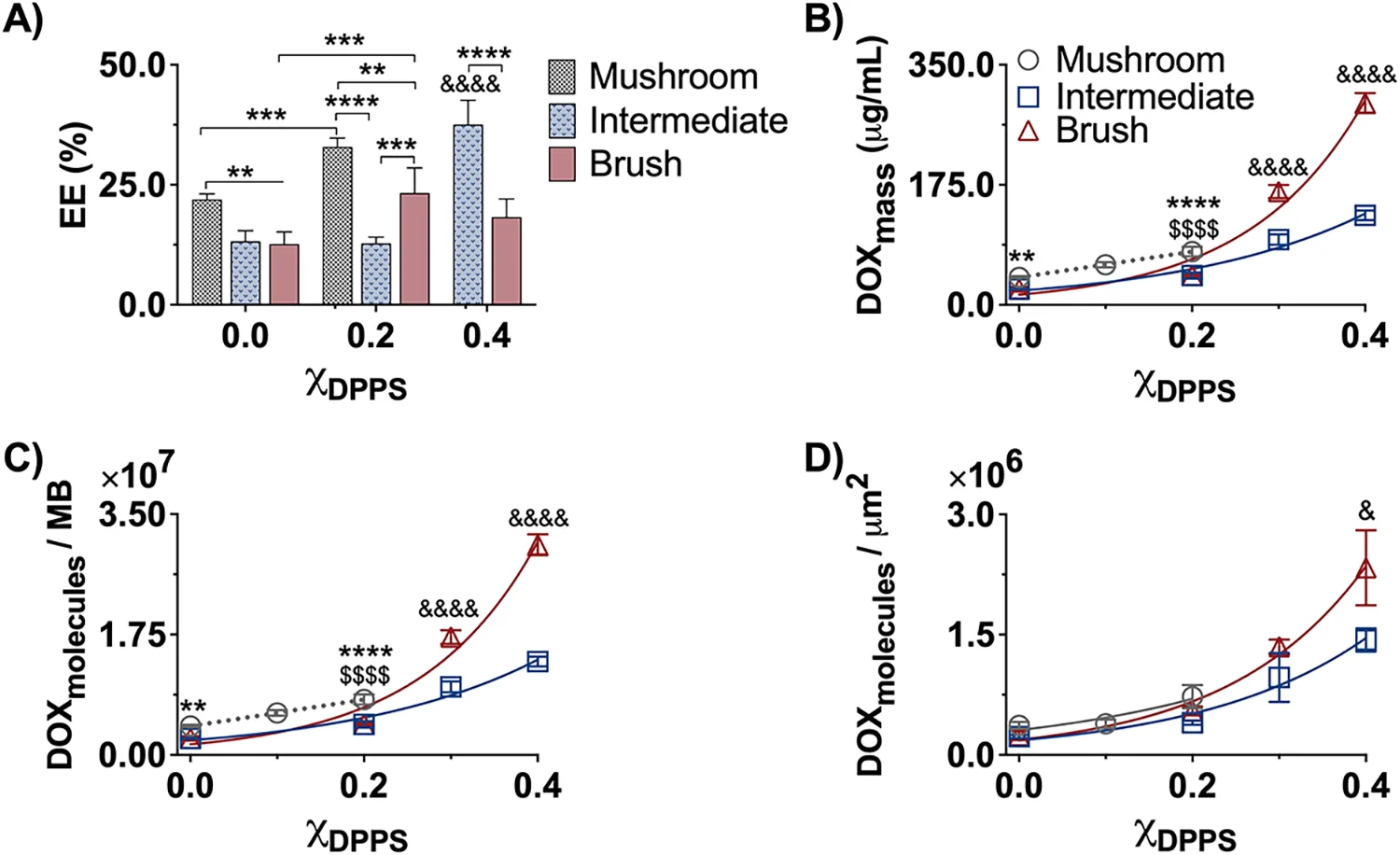
Multiscale quantification of DOX loading across PEG-coated MB architectures. A) Encapsulation efficiency (EE%) as a function of χ_DPPS_ and PEG architecture. Statistical significance is indicated as **^,^ ***^,^ ****p < 0.005; ^&&&&^p < 0.0005 *vs.* intermediate at χ_DPPS_ = 0.2. B–D) Multiscale DOX loading descriptors normalized to 1 × 10^10^ MBs/mL: B) macroscopic (DOX_mass_), (C) mesoscopic (DOX_molecules_/MB), and (D) microscopic (DOX_molecules_/μm^2^). Dotted lines represent linear fits (R^2^ > 0.95), and solid lines represent exponential fits (R^2^ > 0.90). Data represent the mean ± standard deviation of five independent experiments. Statistical comparisons at equivalent χ_DPPS_ are indicated as **p < 0.05 (mushroom *vs*. brush), ^$$$$^p < 0.0005 (mushroom *vs*. intermediate), and ^&, &&&&^p < 0.05 (brush *vs*. intermediate).

To decouple bulk loading from interfacial adsorption, DOX encapsulation was quantified using three hierarchical descriptors: macroscopic (DOX_mass_), mesoscopic (DOX_molecules_/MB), and microscopic (DOX_molecules_/μm^2^), with all formulations normalized, to 1 × 10^10^ MBs/mL (Figure 3B–D) to enable direct comparison among formulations while accounting for concentration-dependent differences in MB populations.^1,36^ At the macroscopic level (Figure 3B), DOX_mass_ increased linearly with χ_DPPS_ in mushroom MBs (40.2 ± 2.0 to 77.7 ± 6.9 μg/mL), whereas intermediate and brush architectures exhibited nonlinear increases, reaching 130.5 ± 7.0 and 294.0 ± 14.7 μg/mL at χ_DPPS_ = 0.4, respectively. Similar trends were observed at the mesoscopic level (Figure 3C), where intermediate and brush MBs reached 1.6 ± 0.1 × 10^7^ and 3.0 ± 0.2 × 10^7^ DOX_molecules_/MB, respectively. Together, these macroscopic and mesoscopic descriptors indicate increased association at the formulation and individual MB levels. However, despite normalization to an equivalent number of MBs, both descriptors remain influenced by formulation-dependent differences in accessible interfacial area.

In contrast, microscopic normalization revealed convergence of interfacial DOX loading across architectures, with surface densities of 1.4 ± 0.1 × 10^6^ and 2.3 ± 0.5 × 10^6^ DOX_molecules_/μm^2^ despite substantial differences in EE% and bulk loading (Figure 3D). This convergence indicates that the larger variations observed at the macroscopic and mesoscopic levels primarily arises from differences in accessible interfacial surface area rather than proportional changes in intrinsic adsorption capacity. Thus, the microscopic descriptor (DOX_molecules_/μm^2^) minimizes geometric contributions and enables a more direct comparison of DOX interfacial adsorption behavior among PEG architectures. Importantly, this metric can be readily calculated from EE% by normalizing the encapsulated DOX to a common number of MBs and accounting for their total surface area, providing a straightforward and adaptable complementary basis for more objective and rational formulation selection based on interfacial DOX loading.

Surface-normalized descriptors are increasingly recognized as the most physically relevant metrics in lipid-based delivery systems, because interfacial density, rather than bulk concentration, governs functional performance.^51,52^ This distinction is particularly important in MB systems, where identical MB concentrations can exhibit different accessible surface areas due to variations in size distributions. Consistent with these framework, our previous study demonstrated that mesoscopic descriptors can overestimate molecular loading relative to surface-normalized metrics,^5^ whereas microscopic normalization provides a robust basis for comparing MB formulations while accounting for size- and concentration-dependent differences.^5,36^ Moreover, because spatial organization of DOX within the lipid shell can directly influences acoustic behavior,^18^ interfacial organization may represent an important determinant of MB functionality beyond total drug payload. While the microscopic descriptor establishes differences in DOX surface density among the evaluated formulations, whether these differences influence US-triggered drug release, cavitation behavior, and ultimately therapeutic performance remain to be determined. Determining this relationship will clarify the functional relevance of DOX surface density in drug-loaded MBs.

### 3.3 Thermodynamic Analysis of DOX Adsorption

#### 3.3.1 Apparent Free Energy of Adsorption (ΔG_app_)

To further evaluate the energetics of DOX loading, ΔG_app_ was estimated from the K_app_ values derived from the loading descriptors presented in Figure 3 B–D (Figure S4). Because the adsorption profiles did not exhibit a classical saturation plateau, K_app_ was treated as an apparent operational descriptor rather than an equilibrium adsorption constant. ΔG_app_ was calculated at the macroscopic (ΔG_app, mass_), mesoscopic (ΔG_app, MB_), and microscopic (ΔG_app, SA_) levels using the same

reference MB concentration (C_ref_ =1 × 10^10^ MBs/mL). The K_app_ values exhibited a clear dependence on lipid composition and PEG architecture (Figure S4A). Increasing PEG density reduced K_app_ at a given χ_DPPS_, indicating decreased apparent DOX accessibility associated with increasing steric crowding, whereas increasing χ_DPPS_ increased K_app_ across all architectures, consistent with enhanced interactions between DOX and the anionic DPPS sites. The maximum K_app_ values were 0.16 ± 0.01, 0.25 ± 0.03, and 0.60 ± 0.03 for mushroom, intermediate, and brush architectures, respectively.

Accordingly, ΔG_app_ values exhibited the same architecture-and composition-dependent trends observed for K_app_ (Figure S4B). The consistent variations in ΔG_app, mass_, ΔG_app, MB_, ΔG_app, SA_ indicates that PEG architecture and lipid composition modulate a common interfacial adsorption process across different loading descriptors. At low χ_DPPS_ (0.0–0.2), mushroom MBs exhibited lower ΔG_app_ values (6.3 ± 0.1 and 4.6 ± 0.2 kJ/mol) than intermediate (7.6 ± 0.1 and 6.1 ± 0.3 kJ/mol) and brush (7.7 ± 0.4 and 6.1 ± 0.1 kJ/mol) MBs, consistent with reduced steric constraints associated with lower PEG grafting densities.^50^ Increasing χ_DPPS_ (0.3–0.4), further decreased ΔG_app_ for intermediate (4.1 ± 0.2 and 3.5 ± 0.3 kJ/mol) and brush (2.8 ± 0.1 and 1.3 ± 0.1 kJ/mol) MBs. These positive ΔG_app_ values are consistent with K_app_ < 1,^53^ whereas the progressive decrease in ΔG_app_ reflects increasingly favorable apparent adsorption energetics.^13,14^ This behavior suggests that although dense PEG layers may initially restrict DOX accessibility, the lateral heterogeneity of lipid-coated MB interfaces, characterized by phospholipid-rich domains and polymer-rich regions,^31^ may create localized interfacial environments that promote cooperative DOX accumulation.

Although ΔG_app_ captures the overall energetic of DOX loading, it does not resolve the molecular interactions responsible for the observed adsorption behavior. Therefore, the relative contributions of the intermolecular interactions governing DOX adsorption were quantified to elucidate the molecular origin of ΔG_app_ (Figure 4A). Previous studies have shown that DOX adsorption at phospholipid interfaces involves hydrophobic interactions with lipid hydrocarbon chains and electrostatic interactions with anionic phospholipids and DSPE-mPEG_2000_,^17,18,20,54^ whereas DOX self-association occurs through π–π stacking interactions between anthracycline rings.^40^ At χ_DPPS_ = 0.0, the relative contribution of hydrophobic interactions decreased from 94.1 ± 1.0% to 9.6 ± 13.3% as PEG density increased from mushroom to brush architectures (Figure 4Aa), consistent with an increase in the contribution of negatively charged DSPE-mPEG_2000_ (5.9 ± 1.0% to 90.4 ± 13.3%).^54^ Upon incorporation of DPPS (χ_DPPS_ = 0.2), electrostatic interactions became the dominant contribution across all architectures, accounting for 76.3 ± 1.0% and 96.1 ± 4.3% of the total interaction profile (Figure 4Ab). At χ_DPPS_ = 0.3–0.4, intermediate architectures remained predominately governed by electrostatic interactions (82.0 ± 19.6% and 71.6 ± 7.5%), accompanied by an increased contribution from DOX–DOX π–π interactions (11.9 ± 14.2% and 20.1 ± 7.5%). In contrast, brush architectures exhibited increasingly balanced electrostatic (67.7 ± 5.5% and 50.0 ± 9.0) and DOX–DOX π–π contributions (28.5 ± 5.7% and 50.5 ± 8.1) (Figure 4Ab, c). This redistribution of intermolecular interactions is consistent with membrane model observations proposing a two-step DOX binding mechanism at anionic phospholipid interfaces, involving initial electrostatic association of the dihydroxyanthraquinone moiety at the membrane surface followed by partial hydrophobic insertion into the lipid interface.^55^ The relatively small hydrophobic contribution in intermediate and brush architectures is consistent with the low hydrophobicity of DOX together with the conformation-dependent hydrophobic shielding imposed by PEG chains, which is greatest in brush architectures, and the decreasing fraction of neutral lipids available for hydrophobic interactions as negatively charged phospholipid content increases.^39,50^ Thus, once DOX is adsorbed at the MB interface, additional DOX loading can be associated with DOX–DOX π–π stacking interactions.^12,17,18^

**Figure 4:**
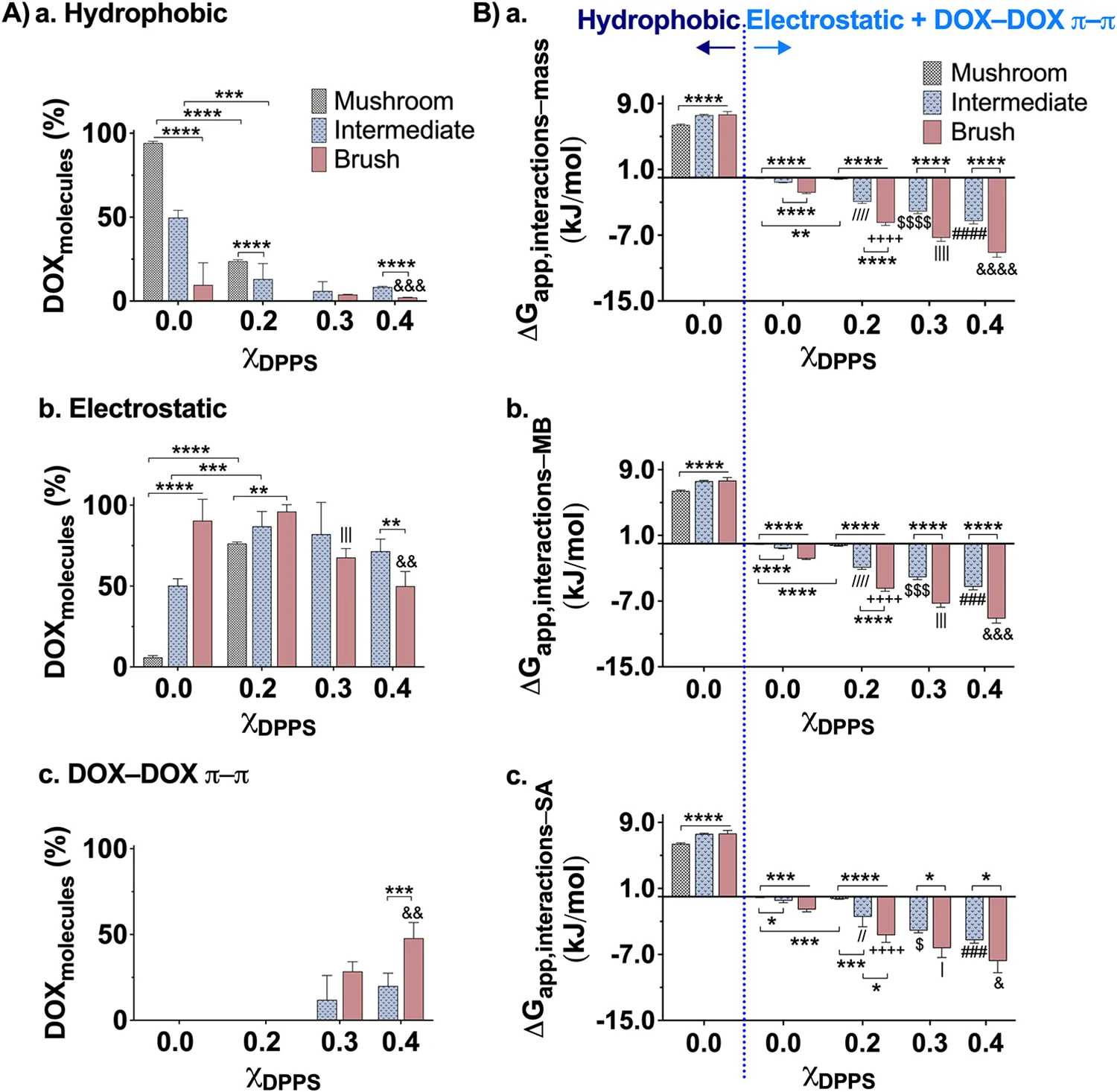
Molecular interaction contributions and apparent thermodynamic analysis of DOX adsorption onto PEG-coated MB architectures. A) Relative contributions of (a) hydrophobic, (b) electrostatic, and (c) DOX–DOX π–π interactions as a function of χ_DPPS_ and PEG density. B) Apparent free energy changes (ΔG_app_) associated with the hydrophobic and combined electrostatic/DOX–DOX π–π adsorption regimes, evaluated using (a) macroscopic (ΔG_app,mass_), (b) mesoscopic (ΔG_app,MB_), and (c) microscopic (ΔG_app,SA_) loading descriptors. ΔG_app_ values were calculated from K_app_ obtained from the corresponding adsorption relationships in Figure 3. Data represent the mean ± standard deviation of five independent experiments. Statistical significance is indicated as *^,^ ***^,^ ****p < 0.05; ^//,///,////^p < 0.005 *vs.* intermediate at χ_DPPS_ = 0.0 electrostatic + DOX–DOX π–π, ^$,$$$,$$$$^p < 0.05 *vs.* intermediate at χ_DPPS_ = 0.2, ^###, ####^p < 0.005 *vs.* intermediate at χ_DPPS_ = 0.3, ^++++^p < 0.0005 *vs.* brush at χ_DPPS_ = 0.0 electrostatic + DOX–DOX π–π, ^|,|||,||||^p < 0.05 *vs.* brush at χ_DPPS_ = 0.2, and ^&,&&,&&&,&&&&^p < 0.05 *vs.* brush at χ_DPPS_ = 0.3.

To determine how these molecular interactions influence the thermodynamic driving force of DOX adsorption, their energetic contributions were subsequently evaluated across the loading descriptors (ΔG_app, interactions–mass_, ΔG_app, interactions–MB_, and ΔG_app, interactions–SA_; Figure 4B) using classical interfacial free-energy frameworks, in which adsorption energetics are interpreted as the combined result of multiple molecular contributions associated with hydrophobic and hydrophilic molecular domains (eq 16).^44^ At χ_DPPS_ = 0.0, ΔG_app, interactions–mass_, ΔG_app, interactions–MB_, and ΔG_app, interactions–SA_ were positive across all PEG architectures and increased from 6.3 ± 0.1 kJ/mol for mushroom MBs to 7.7 ± 0.4 kJ/mol brush MBs. This increase coincided with the reduced relative contribution of hydrophobic interactions at higher PEG densities, suggesting that PEG-induced steric constraints limit access to hydrocarbon lipid domains involved in DOX association.^39,50^ Consequently, the overall adsorption process remained endergonic,^13,53^ indicating that the favorable contribution of hydrophobic interactions was insufficient to compensate for the energetic costs associated with PEG organization and local interfacial rearrangement. Positive adsorption free-energy values have been reported for systems where adsorption is primarily influenced by hydrophobic interactions.^56^

Increasing χ_DPPS_ (0.2–0.4), progressively shifted ΔG_app, interactions_ toward more negative values across all PEG architectures (Figure 4B). The ΔG_app, interactions–mass_ and ΔG_app, interactions–MB_ descriptors decreased from −0.30 ± 0.05 kJ/mol to −5.50 ± 0.30 kJ/mol at χ_DPPS_ =0.2, and from −4.70 ± 0.30 kJ/mol to −7.70 ± 0.90 kJ/mol at χ_DPPS_ = 0.4 as PEG architecture transitioned toward brush regimes. In contrast, ΔG_app, interactions–SA_ exhibited a narrower variation, decreasing from −1.90 ± 0.80 kJ/mol to −3.10 ± 0.60 kJ/mol at χ_DPPS_ = 0.2, and from −5.3 ± 0.40 kJ/mol to −9.1 ± 0.50 kJ/mol at χ_DPPS_ = 0.4. These changes coincided with the increasing contribution of electrostatic and DOX–DOX π–π interactions observed in Figure 4Ab–c, suggesting an increasingly exergonic adsorption process driven by stronger attractive interactions that favor interfacial DOX retention.^13,53^ Consistently, K_app_, _interactions_ > 1 were observed under these conditions (Figure S5), with values ranging from 1.9 ± 0.24, 9.4 ± 1.50, and 19.0 ± 5.2 for mushroom, intermediate, and brush architectures, respectively. Thus, consistent with the negative ΔG_app, interactions_ values obtained and support a favorable apparent adsorption process.^53^ Negative adsorption free-energy values have likewise been reported for DOX adsorption onto nanostructured and carbohydrate-based interfaces.^57^

These results demonstrate that ΔG_app_ represents the net balance of multiple molecular interactions, whose individual contributions may be masked within the overall energetic profile. Similar free-energy decomposition approaches in molecular association systems have shown that resolving individual energetic components provides mechanistic insight beyond the overall free-energy value.^58,59^ Moreover, molecular dynamics studies have demonstrated that the free-energy landscape governing DOX association with phospholipid interfaces is strongly dependent on lipid composition and membrane packing, which regulate the thermodynamic favorability of interfacial DOX binding.^60^ Consequently, resolving these contributions enabled the identification of the energetic factors underlying formulation-dependent DOX adsorption at the PEGylated lipid MB shell.

The lower variability of ΔG_app, interactions–SA_ indicates that expressing adsorption energetics per interfacial area reduces the influence of MB size distribution, enabling more meaningful comparisons among formulations. Therefore, ΔG_app, interactions–SA_ was selected as the most representative descriptor of apparent DOX adsorption energetics. Unlike EE%, which reflects the overall amount of DOX incorporated into the MB population, ΔG_app, interactions–SA_ provides insight into the energetic basis of DOX–MB association. The distinct trends between these descriptors demonstrate that the formulation exhibiting the highest DOX loading does not necessarily correspond to the one with the most favorable adsorption energetics, highlighting that the overall DOX incorporation depends on the combined effects of interfacial affinity, PEG/lipid organization, and available surface area.

#### 3.3.2 Apparent Enthalpic (ΔH_app, SA_) and Entropic (ΔS_app, SA_) Contributions

The interaction-based analysis presented above provided mechanistic insight into the molecular factors governing DOX adsorption at the MB interface; however, it does not resolve the overall thermodynamic driving forces associated with the apparent adsorption process. Therefore, temperature-dependent experiments were performed across PEG architectures and χ_DPPS_ conditions under identical loading conditions (0.5 mg/mL DOX and 10 min incubation) to determine ΔH*_app, SA_* and ΔS*_app, SA_* contributions governing DOX adsorption. The evaluated temperatures (277.15, 298.15, and 310.15 K) represent relevant storage, handling, and physiological conditions for DOX–MBs.

Temperature-dependent variations in DOX–MB concentration were observed, with differences dependent on PEG architecture and lipid composition (Figure S6). Relative to 298.15 K, DOX–MB concentrations decreased at 277.15 and 310.15 K by approximately 53 ± 13%, 59 ± 5% and 56 ± 10% for mushroom, intermediate, and brush architectures, respectively, with the magnitude of these changes varying with χ_DPPS_. These results suggest that temperature influences MB retention, likely through changes in lipid monolayer organization and interfacial rearrangements.^20,61^ Additionally, these variations highlight the importance of normalizing adsorption process measurements using a common reference concentration (1 × 10^10^ MBs/mL),^1,36^ enabling direct comparisons of interfacial adsorption behavior across temperatures, PEG architectures, and lipid compositions.

Accordingly, DOX adsorption was quantified using the microscopic descriptor (DOX_molecules_/μm^2^). Surface loading exhibited architecture-, composition-, and temperature-dependent profiles (Figure S7A). Intermediate and brush MBs displayed nonlinear increases in surface loading with increasing χ_DPPS_, reaching maximum values of 1.4–2.5 × 10^6^ and 2.4–5.5 DOX_molecules_/μm^2^, respectively. In contrast, mushroom MBs exhibited predominantly linear adsorption profiles at 277.15 and 310.15 K, whereas a nonlinear response was observed at 298.15 K, reaching lower surface densities (1.5 ± 0.2, 2.6 ± 0.2 and 0.7 ± 0.1 × 10^6^ DOX_molecules_/μm^2^). These adsorption profiles were subsequently used to determine K_app, interactions–SA_ values for thermodynamic analysis.

Increasing temperature reduced K_app, interactions–SA_ values across formulations, whereas increasing χ_DPPS_ and PEG grafting density increased K_app, interactions–SA_ at each evaluated temperature (Figure S7B). K_app,_ _interactions–SA_ < 1 was generally observed at χ_DPPS_= 0.0–0.2, whereas K_app,interactions–SA_ > 1 was predominantly observed for brush MBs at χ_DPPS_= 0.3–0.4. Although 277.15 and 310.15 K increased apparent DOX surface loading, the associated decrease in MB concentration indicates a trade-off between adsorption efficiency and MB retention (Figure S6). Therefore, 298.15 K provided the most favorable balance between DOX adsorption and MB concentration under the evaluated conditions. The dependence of K_app, interactions–SA_ on PEG architecture and lipid composition indicates that adsorption affinity is governed not only by the availability of lipid binding sites but also by the physicochemical properties of the interface. Previous studies have shown that interfacial hydration can modulate drug–lipid association by altering the energetic balance of adsorption.^62^ Accordingly, the higher *K*_app, interactions–SA_ values observed for PEG-dense architectures at higher χ_DPPS_ may result from a more favorable interfacial environment that promotes DOX association through electrostatic interactions and DOX–DOX π–π stacking.

To further characterize the thermodynamic basis of this temperature-dependent adsorption behavior, a van’t Hoff analysis of ln­(K_app, interactions–SA_) as a function of 1/T was performed (Figure 5Aa). Distinct PEG architecture-dependent profiles were observed. Mushroom MBs exhibited biphasic response, with a positive slope between 277.15 and 298.15 (R^2^ = 1.0), and a negative slope between 298.15 and 310.15 K (R^2^ = 1.0). No statistically significant changes were observed between 277.15 and 298.15 K, whereas a significant decrease was observed between 298.15 and 310.15 K. Intermediate MBs exhibited minimal temperature dependence across the evaluated range (R^2^ = 0.97), whereas brush MBs displayed the greatest temperature sensitivity, primarily between the lowest and highest temperatures evaluated (R^2^ = 0.98). The biphasic response observed for mushroom MBs suggests that sparsely grafted PEG layers exhibit a distinct temperature response compared with higher grafting densities. This behavior may be associated with greater conformational adaptability of PEG chains at low grafting densities. PEG/PEO chains exhibit temperature-dependent conformational behavior in aqueous media, influenced by the balance between polymer hydration and chain organization.^63,64^ Therefore, differences in PEG grafting density may regulate the extent of temperature-induced interfacial changes, resulting in distinct adsorption regimes.

**Figure 5:**
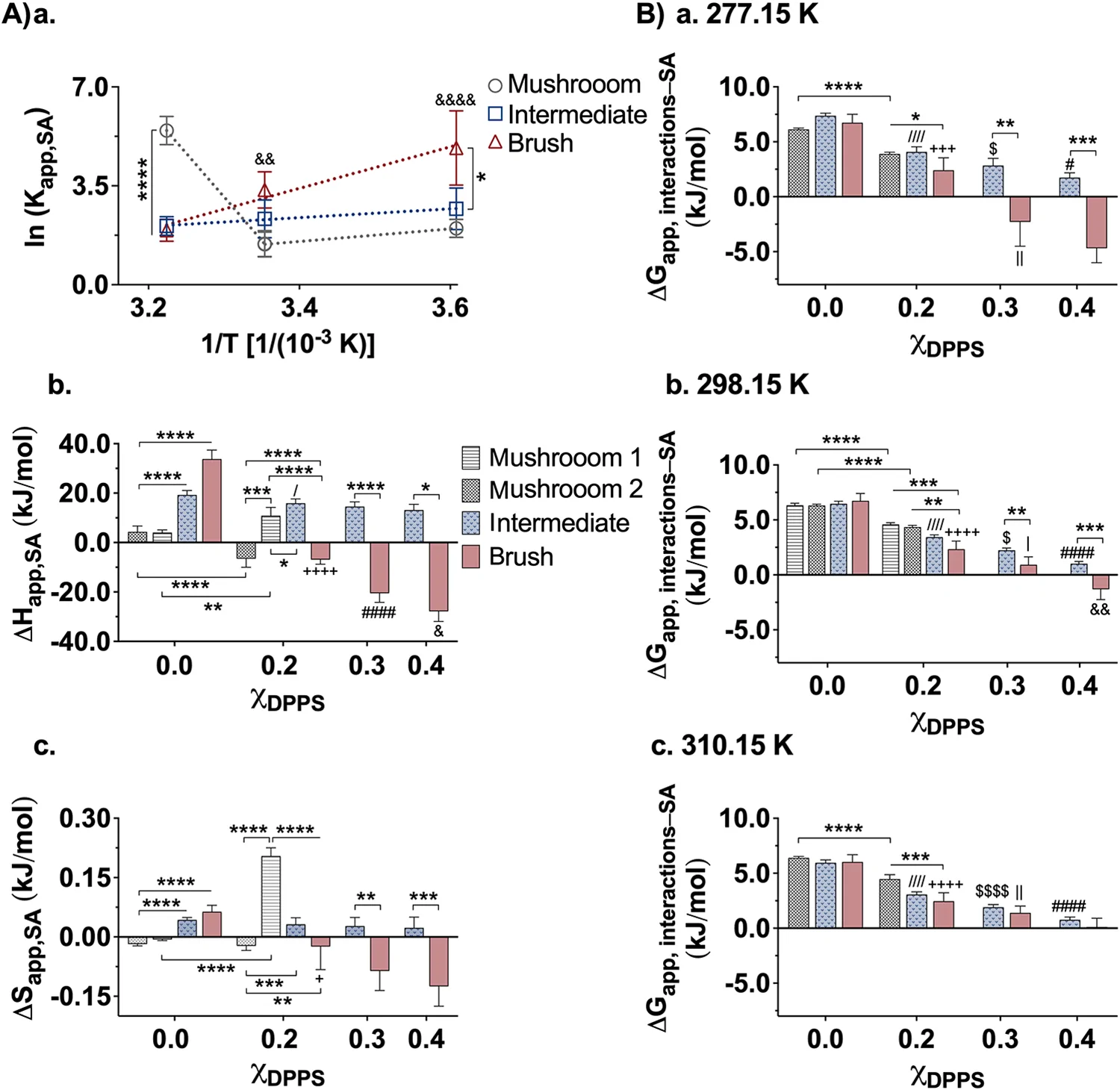
Temperature-dependent thermodynamic analysis of DOX adsorption at PEGylated MB interfaces. A) Temperature-dependent thermodynamic parameters obtained from the adsorption analysis: (a) Van’t Hoff plots of ln (K_app, interactions–SA_) versus 1/T. Dotted lines represent linear fits with (R^2^ > 0.98). Statistical significance is indicated as ****p < 0.0005, and ^&&^p < 0.005 (mushroom *vs.* brush). Corresponding (b) apparent enthalpic (ΔH_app, SA_) and (c) entropic (ΔS_app, SA_) contributions. Mushroom 1 (298.15 to 310.15 K) and Mushroom 2 (277.15–298.15 K). Statistical significance is indicated as *^,^ **^,^ ***^,^ ****p < 0.05; ^|^p < 0.05 *vs.* brush at χ_DPPS_ = 0.2, and ^&^p < 0.05 *vs.* brush at χ_DPPS_ = 0.3. B) Apparent adsorption free energy (ΔG_app_,_interactions–SA_) calculated from the enthalpic and entropic contributions (ΔG_app,interactions–SA_= ΔH_app, SA_–TΔS_app, SA_) at (a) 277.15 K, (b) 298.15 K, and (c) 310.15 K. Statistical significance is indicated as ^*, **, ***, ****^p < 0.05; ^|^p < 0.05 *vs.* brush at χ_DPPS_ = 0.2, and ^&^p < 0.05 *vs.* brush at χ_DPPS_ = 0.3. Data represent the mean ± standard deviation of five independent experiments.

The ΔH_app, SA_ and ΔS*_app, SA_* values were subsequently derived from the slope (m = −ΔH_app,SA_/R) and intercept (b = ΔS_app,SA_/R) of the van’t Hoff plots, respectively. According to the van’t Hoff relationship, positive slopes correspond to exothermic (ΔH_app,_ _SA_ < 0) processes, whereas negative slopes indicate endothermic processes (ΔH_app,_ _SA_ > 0).^45,53^ Positive ΔH_app, SA_ values were observed for all PEG architectures at χ_DPPS_ = 0.0–0.3 and progressively decreased with increasing χ_DPPS_ (Figure 5Ab). Specifically, ΔH_app,SA_ increased from 4.3 ± 2.4 kJ/mol to 10.8 ± 3.4 kJ/mol for mushroom MBs (Mushroom 1, 298.15 to 310.15 K) and decreased from 19.3 ± 1.8 kJ/mol to 14.5 ± 1.9 kJ/mol for intermediate MBs, and from 33.8 ± 3.6 kJ/mol to −20.5 ± 3.7 kJ/mol for brush MBs. At χ_DPPS_ = 0.4, ΔH*_app, SA_* was positive for intermediate (13.1 ± 2.4 kJ/mol) and became negative for brush MBs (−27.8 ± 4.1 kJ/mol). For mushroom MBs, the 277.15–298.15 K regime (Mushroom 2) yielded a negative ΔH*_app, SA_* value (−6.5 ± 3.47 kJ/mol).

Overall, ΔS*_app, SA_* values were small in magnitude across all conditions (on the order of 10^–2^ kJ/mol) (Figure 5Ac). Nevertheless, architecture- and composition-dependent sign changes were observed. Mushroom MBs exhibited predominantly negative ΔS*_app, SA_* values, whereas intermediate and brush MBs displayed positive values at low χ_DPPS_ that shifted toward negative values with increasing χ_DPPS_. Increasing PEG grafting density above χ_PEG_ = 0.05 has been reported to promote lipid headgroup dehydration, enhanced lateral packing of lipid acyl chains, and hydration of the outer PEG layer, reducing interfacial defects and increasing PEGylated interface stability.^64^ These structural rearrangements impose conformational constraints within the PEG layer, contributing to the unfavorable ΔS*_app, SA_* observed during adsorption.^65^ The favorable ΔS*_app, SA_* contributions observed for intermediate and brush architectures at χ_DPPS_ = 0.4 may therefore reflect stabilizing DOX–interface interactions that compensate for ΔS*_app, SA_* penalty associated with PEG-mediated organization.^62^

The combined ΔH_app, SA_ and ΔS*_app, SA_* contributions were subsequently used to calculate ΔG_app, interactions–_ *_SA_* according to eq 17 (Figure 5B). Across all temperatures, ΔG_app, interactions–_ *_SA_* decreased with increasing χ_DPPS_ from 0.0 to 0.3 for all PEG architectures. At χ_DPPS_ = 0.4, intermediate MBs exhibited the lowest ΔG_app, interactions–_ *_SA_* values at 277.15 and 298.15 K (1.45 ± 0.7 kJ/mol and 1.01 ± 0.2 kJ/mol, respectively), whereas brush MBs displayed negative ΔG_app, interactions–_ *_SA_* under these conditions (−3.1 ± 2.4 kJ/mol and −1.3 ± 0.9 kJ/mol, respectively). The magnitude of ΔG_app, interactions–_ *_SA_* was consistent with the trends observed for *K*_app, interactions–SA_ and with the net apparent free-energy contributions associated with the different adsorption interaction regimes (Figure 4, eq 16), supporting the thermodynamic framework proposed for DOX adsorption at the MB interface. The favorable ΔG_app, interactions–_ *_SA_* values obtained at 298.15 K, combined with the higher MB concentration retained at this temperature, supported its selection for DOX–MB formulation.

### 3.4 Thermodynamically Guided Optimization of Interfacial Loading

Guided by the thermodynamic framework, DOX loading was optimized by varying incubation time and drug concentration in intermediate and brush architectures at χ_DPPS_ = 0.0 and 0.4 (Figure 6). Mushroom MBs were excluded because of instability under highly charged and prolonged incubation conditions (>10 min at χ_DPPS_ = 0.2), limiting their suitability for high-payload formulations.

**Figure 6:**
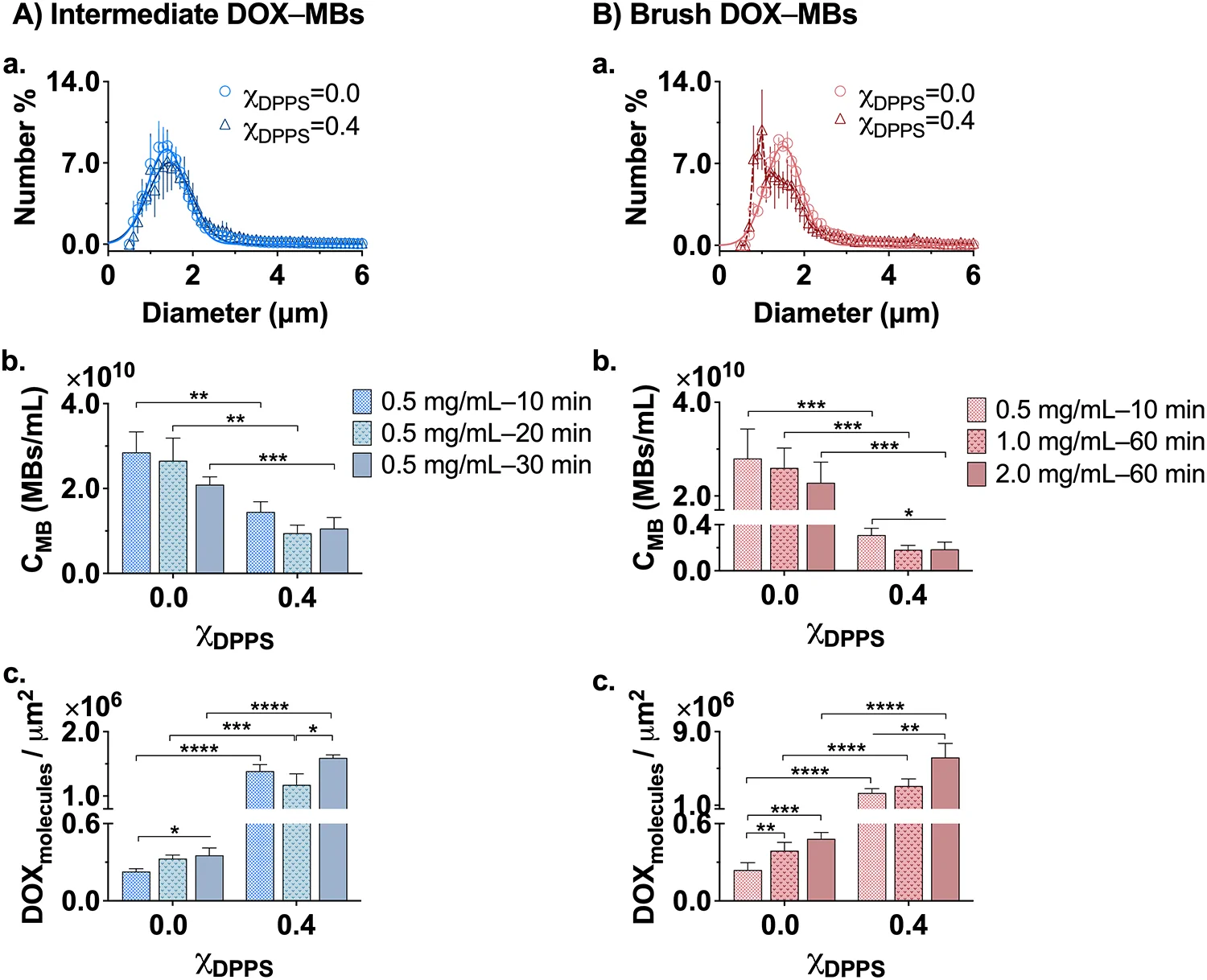
Maximization of interfacial DOX loading under extended incubation and concentration conditions. (A) Intermediate and (B) brush architectures evaluated at χ_DPPS_ = 0.0 and 0.4. (a) Number-weighted size distributions; shaded regions denote standard deviation (SD). Solid lines represent Gaussian fits (R^2^ ≥ 0.95), and dashed lines indicate point-to-point connections. (b) Total microbubble concentration (C_MB_) and (c) interfacial loading expressed as DOX_molecules_/μm^2^, normalized to 1 × 10^10^ MBs/mL. Statistical significance is indicated as *p < 0.05. Data represent the mean ± standard deviation of five independent experiments.

Number-weighted size distributions (Figure 6Aa) exhibited-well-defined populations across all evaluated conditions and were described by Gaussian fits (R^2^ ≥ 0.95). In the brush architectures, incorporation of DPPS shifted MB populations toward smaller diameters relative to χ_DPPS_ = 0.0, resulting in D_m_ of ∼1.7 ± 0.8 μm (Figure S8Ab) and SA normalized to 1 × 10^10^ MBs/mL of ∼1.1 ± 0.2 × 10^11^μm^2^ (Figure S8Ac). Correspondingly, C_MB_ (Figure 6Ab–Bb) decreased in the presence of DPPS, consistent with increased interfacial electrostatic stress in highly anionic formulations. At χ_DPPS_ = 0.0, C_MB_ reached 2.5 ± 0.5 × 10^10^ MBs/mL, whereas χ_DPPS_ = 0.4 reduced concentrations to 1.2 ± 0.3 × 10^10^ MBs/mL for intermediate MBs and 2.3 ± 0.8 × 10^9^ MBs/mL for brush MBs. However, when normalized to 1 × 10^10^ MBs/mL, the accessible SA increased to ∼1.5 ± 0.5 × 10^11^μm^2^ (Figure S8Bc).

Interfacial loading (Figure 6A) revealed distinct architecture-dependent optimization windows. In intermediate MBs, loading remained comparable between 10- and 20 min incubation (∼1.3 ± 0.2 × 10^6^ DOX_molecules_/μm^2^) using 0.5 mg/mL DOX, increasing to 1.6 ± 0.04 × 10^6^ DOX_molecules_/μm^2^ at 30 min. However, extending incubation beyond 30 min or increasing DOX concentration to 1.0 mg/mL induced MB agglomeration (Figure S9), indicating that loading capacity was constrained by limited colloidal stability under elevated interfacial stress. This behavior can be explained by the larger PEG chain dimensions and increased chain extension relative to mushroom MBs (Table 1), which provide greater steric accommodation for DOX within the PEG layer. Therefore, higher interfacial DOX densities are required before adsorbed DOX molecules become sufficiently exposed to promote DOX–DOX π–π association between neighboring MBs. As a result, aggregation is progressively favored with prolonged incubation or increased DOX concentration as the interfacial DOX accumulation increases (Figure 2B).

In contrast, brush architectures remained colloidally stable across all evaluated conditions (Figure S10) and exhibited substantial increases in interfacial loading with increasing DOX concentration, raising from 3.1 ± 0.8 × 10^6^ at 1.0 mg/mL to 6.2 ± 1.5 × 10^6^ at 2.0 mg/mL without evidence of aggregation. This behavior is consistent with the greater PEG chain length and grafting density of the brush architecture, which provide a larger interfacial volume for DOX incorporation and prevent the critical interfacial DOX density required for intermolecular DOX–DOX π–π stacking between neighboring MBs from being reached under the evaluated conditions.

Although MB concentration decreased with increasing χ_DPPS_, DOX_molecules_/μm^2^ increased because this parameter was calculated after normalizing MB concentration to 1 × 10^10^ MBs/mL. At this equivalent MB population, the accessible surface area showed a slight increase with χ_DPPS_, while the amount of adsorbed DOX increased to a greater extent, resulting in higher DOX surface density. This indicates that the enhanced loading arises from stronger DOX–MB interfacial interactions rather than from differences in MB concentration.

These findings support the thermodynamic framework, demonstrating that architectures exhibiting more favorable ΔG_app, interactions–SA_ and ΔH_app,SA_ values not only support higher interfacial loading but also provide expanded colloidal stability window for high-payload formulations. All together, these results demonstrate that optimization of DOX–MB formulations are governed by the underlying interfacial energetics that regulate both adsorption capacity and colloidal stability, rather than by empirical loading conditions alone.

### 3.5 Comparison of Interfacial Loading and Interaction Contributions across PEG Architectures

Integration of the optimal architecture-specific conditions revealed a hierarchical increase in maximal interfacial DOX loading across PEG conformations (Figure 7A). Mushroom MBs exhibited the lowest surface density, reaching 8.0 ± 0.7 × 10^5^ DOX_molecules_/μm^2^ (77.7 ± 6.9 μg/mL at χ_DPPS_ = 0.2, 0.5 mg/mL DOX, 10 min), followed by intermediate MBs with 1.6 ± 0.04 × 10^6^ DOX_molecules_/μm^2^ (182.6 ± 9.9 μg/mL; χ_DPPS_ =0.4, 0.5 mg/mL DOX, 30 min). In contrast, brush architectures produced an increase in loading, reaching 6.2 ± 1.5 × 10^6^ DOX_molecules_/μm^2^ (1017.3 ± 77.7 μg/mL; χ_DPPS_ =0.4, 2.0 mg/mL DOX, 60 min). These findings demonstrate that PEG organization is not merely a steric modifier but as a dominant regulator of interfacial drug adsorption capacity. The increased loading was accompanied by a progressive shift in the dominant interaction mechanisms governing adsorption (Figure 7B). In mushroom MBs, adsorption was primarily driven by hydrophobic interactions (56.0 ± 4.9%), with secondary electrostatic contributions (41.7 ± 3.4%). Transition to the intermediate regime shifted the interaction landscape toward electrostatic dominance (77.1 ± 20.9%) together with the emergence of π–π interactions (15.3 ± 14.2%). In contrast, brush architectures exhibited adsorption predominantly governed by π–π interactions (78.9 ± 5.12%), whereas electrostatic (19.0 ± 2.4%) and hydrophobic contributions (2.9 ± 0.2%) became comparatively minor.

**Figure 7:**
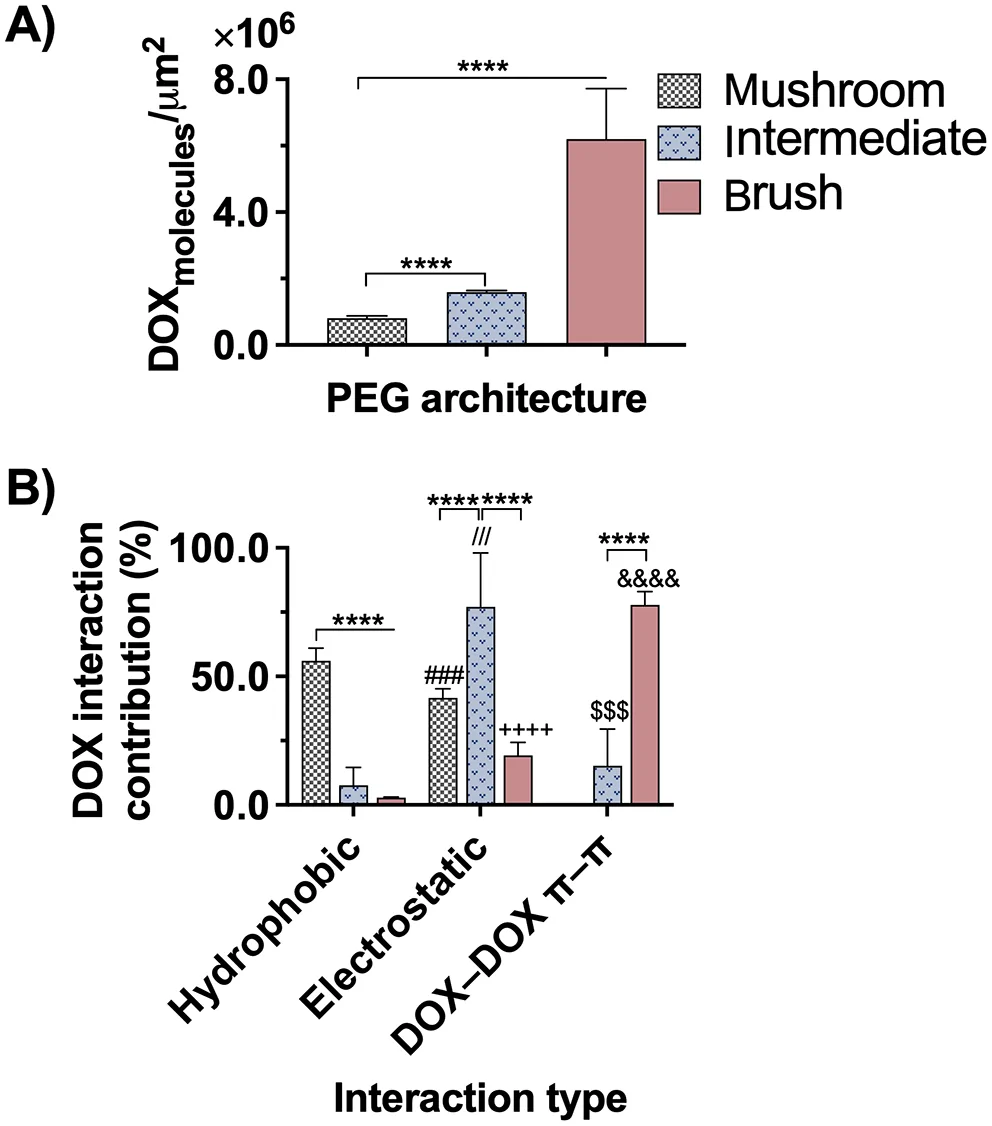
Architecture-dependent DOX loading and interaction contributions. (A) Maximum interfacial DOX loading for mushroom, intermediate, and brush PEGylated MBs, expressed as DOX_molecules_/μm^2^ and normalized to 1 × 10^10^ MBs/mL, under optimal conditions: mushroom (χ_DPPS_ = 0.2, 0.5 mg/mL DOX, 10 min), intermediate (χ_DPPS_ = 0.4, 0.5 mg/mL DOX, 30 min), and brush (χ_DPPS_ = 0.4, 2.0 mg/mL DOX, 60 min). (B) Relative contribution (%) of hydrophobic, electrostatic, and DOX–DOX π–π interactions to DOX adsorption across PEG architectures. Statistical significance is indicated as ****p < 0.0005; ^####^p < 0.0005 *vs.* mushroom (hydrophobic); ^///^p < 0.0005 *vs.* intermediate (hydrophobic); and ^++++^p < 0.0005 vs. brush (hydrophobic), and ^&&&&^p < 0.0005 *vs.* brush (electrostatic). Data represent the mean ± standard deviation of five independent experiments.

Although the relative contributions of hydrophobic, electrostatic, and π-related interactions are inherently system-dependent, molecular simulations of lipid interfaces have shown that adsorption is governed by a coupled free-energy landscape in which interfacial hydration structure plays a central role in modulating the balance of these interactions.^66^ Here, we extend this framework by demonstrating that PEG architecture strongly regulates both interfacial accessibility and the hierarchy of interactions driving DOX adsorption. Although the dense PEG layer in the brush architecture limits DOX diffusion toward the lipid interface through excluded-volume effects, this restriction is overcome as χ_DPPS_ increases. In the absence of negatively charged lipids, reduced hydrophobic interactions indicate limited DOX access to the phospholipid monolayer. However, higher χ_DPPS_ promotes electrostatic attraction and DOX–DOX π–π interactions, which dominate interfacial accumulation. Thus, despite PEG-mediated steric constraints, favorable electrostatic interactions and time-dependent DOX diffusion enable enhanced loading at the brush interface.

Because DOX loading is mediated through non-covalent interactions, interfacial retention remains inherently reversible and susceptible to leakage.^2,67^ Consequently, interaction strength becomes a critical determinant of payload stability. In mushroom MBs, hydrophobic-dominated adsorption was associated with less favorable ΔG_app, SA_ values, indicating weaker interfacial stabilization and limited retention capacity, thereby challenging the use of low PEG density MBs for maximizing DOX loading.^12,18^ In contrast, intermediate and brush architectures exhibited progressively more favorable ΔG_app, SA_ together with enhanced loading, consistent with stronger intermolecular stabilization and improved retention,^17,19,68^ thus supporting their use in therapeutic applications. Notably, π–π interactions have been reported to stabilize DOX-loaded nanosystems by preserving structural integrity upon dilution, improving circulation bioavailability, and enhancing tumor accumulation.^69^ Accordingly, brush architectures combined with the most favorable ΔG_app, SA_ values, highest loading capacity, and dominant π–π interactions, emerges as the most effective configuration for maximizing interfacial drug retention.

Importantly, previously reported DOX payloads for intermediate and brush MB systems typically range from ∼350–750 μg,^17,19^ whereas optimized mushroom-based formulations can reach ∼1400–2500 μg.^12,18^ However, these increases are frequently achieved using formulation-dependent strategies involving organic solvents or elevated temperatures, which may compromise drug integrity and limit clinical translatability. In contrast, the payloads achieved here were obtained using medical-grade DOX under mild aqueous conditions and supported by a thermodynamic framework that directly linking PEG architecture to adsorption energetics, enabling rational selection of optimal interfacial configurations rather than empirical optimization of the formulation. These findings also reconcile previously divergent observations regarding PEG density in MB systems. Low PEG-density mushroom regimes have been associated with prolonged circulation and improved performance in neutral MBs,^22^ favoring diagnostic applications. In contrast, higher PEG densities mitigate complement activation in anionic MBs^70^ and, as demonstrated here, enhance interfacial adsorption strength and loading stability. Rather than representing contradictory behaviors, these effects reflect distinct functional requirements. Overall, PEG architecture emerges as an application-specific design parameter that distinguishes imaging-optimized from therapy-optimized MB formulation, providing a framework to investigate how interfacial properties translate into US-triggered drug release, cavitation, and ultimately therapeutic performance.

## 4 Conclusion

In conclusion, optimization of drug-loaded MB formulations has traditionally relied on bulk metrics such as encapsulation efficiency, assuming that greater drug incorporation reflects improved performance. Here, we demonstrate that bulk loading does not necessarily represent the interfacial adsorption behavior governing drug incorporation. By combining MB concentration normalization with multiscale loading analysis, DOX_molecules_/μm^2^ was identified as the most physically relevant descriptor because it directly relates drug loading to the available interfacial area while reducing variability associated with MB concentration, size, and population distribution. Importantly, DOX_molecules_/μm^2^ provides a readily adaptable, complementary metric to conventional bulk parameters such as EE%, enabling a more objective basis for formulation selection based on interfacial DOX loading. Using this framework, PEG architecture and grafting density were shown to modulate apparent adsorption energetics, revealing that formulations with more favorable interfacial interactions do not always correspond to those with the highest EE%. These findings establish a mechanism-based approach for evaluating and optimizing therapeutic MB formulations according to their interfacial adsorption properties rather than empirical bulk loading alone, providing a rational foundation for the development of next-generation MB-based drug delivery platforms. Linking these interfacial properties to US-mediated drug release and cavitation will be an important next step toward establishing how the molecular basis of DOX incorporation translates into the functional performance of drug-loaded MBs.

## Supplementary Material



## Notes

Figure S1: representative optical microscopy images corresponding to one complete counting quadrant of the Neubauer chamber for DOX–MBs containing different DPPS fractions (χ_DPPS_ = 0.0, 0.2, and 0.4) and exhibiting mushroom, intermediate, and brush PEG architectures; Figure S2: characterization of agglomerates formed in DOX–MBs exhibiting the mushroom PEG regime at high χ_DPPS;_ Figure S3: size and surface area distributions of DOX–MBs with different PEG architectures and χ_DPPS;_ Figure S4: multiscale apparent adsorption constants and apparent Gibbs free energies of adsorption for DOX–MBs; Figure S5: temperature-dependent thermodynamic analysis of DOX adsorption contributions; Figure S6: temperature-dependent variations in DOX–MB concentration across PEG architectures; Figure S7: Microscopic DOX loading and apparent adsorption constants (K_app, interactions–SA_) as a function of χ_DPPS_ for mushroom, intermediate and brush PEG architectures at (a) 277.15 K, (b) 298.15 K, and (c) 310.15 K; Figure S8: characterization of intermediate and brush DOX–MB as a function of DOX concentration and incubation time; Figure S9: characterization of DOX–MB agglomerate formation in the intermediate PEG architecture at χDPPS = 0.4; Figure S10: representative neubauer chamber reconstructions of DOX-MBs in the brush PEG architecture at χ_DPPS_ = 0.4 (PDF)

## Abbreviation

PEG: polyethylene glycol
MBs: microbubbles
US: ultrasound
DOX: doxorubicin
DPPC: dipalmitoyl-*sn*-glycero-3-phosphocholine
DPPS: 1,2-dipalmitoyl-*sn*-glycero-3-phospho-l-serine
DSPE-mPEG_2000_: 1,2-distearoyl-*sn*-glycero-3-phosphoethanolamine-N-[methoxy­(polyethylene glycol)-2000]
PBS: phosphate-buffered saline
SD: standard deviation
SA: surface area
EE%: encapsulation efficiency.
