# Supplementary material for: Polyethylene Glycol Architecture Governs Interfacial Thermodynamics and Drug Adsorption in Anionic Lipid Microbubbles
Source: Langmuir. 2026 Sep 12;42(38):28052–67. doi: 10.1021/acs.langmuir.6c03030 (PMC13629680; doi:10.1021/acs.langmuir.6c03030)
Supplement: Supplementary file 1 [file la6c03030_si_001.pdf]

Supporting information

Polyethylene Glycol Architecture Governs Interfacial Thermodynamics and Drug Adsorption in Anionic Lipid Microbubbles

L. Enrique Corona-Martínez,[1,2] Amelia Ríos,[1] and J. Angel Navarro-Becerra[,1,3]*

[1]Cinvestav–Monterrey, Apodaca NL, 66600, Mexico

[2]FCFM–UANL, San Nicolás de los Garza NL, 66451, Mexico

[3]SECIHTI, Mexico City 03940, Mexico

[*]Corresponding author: J. Angel Navarro-Becerra, PhD, Email: jnavarrob@cinvestav.mx

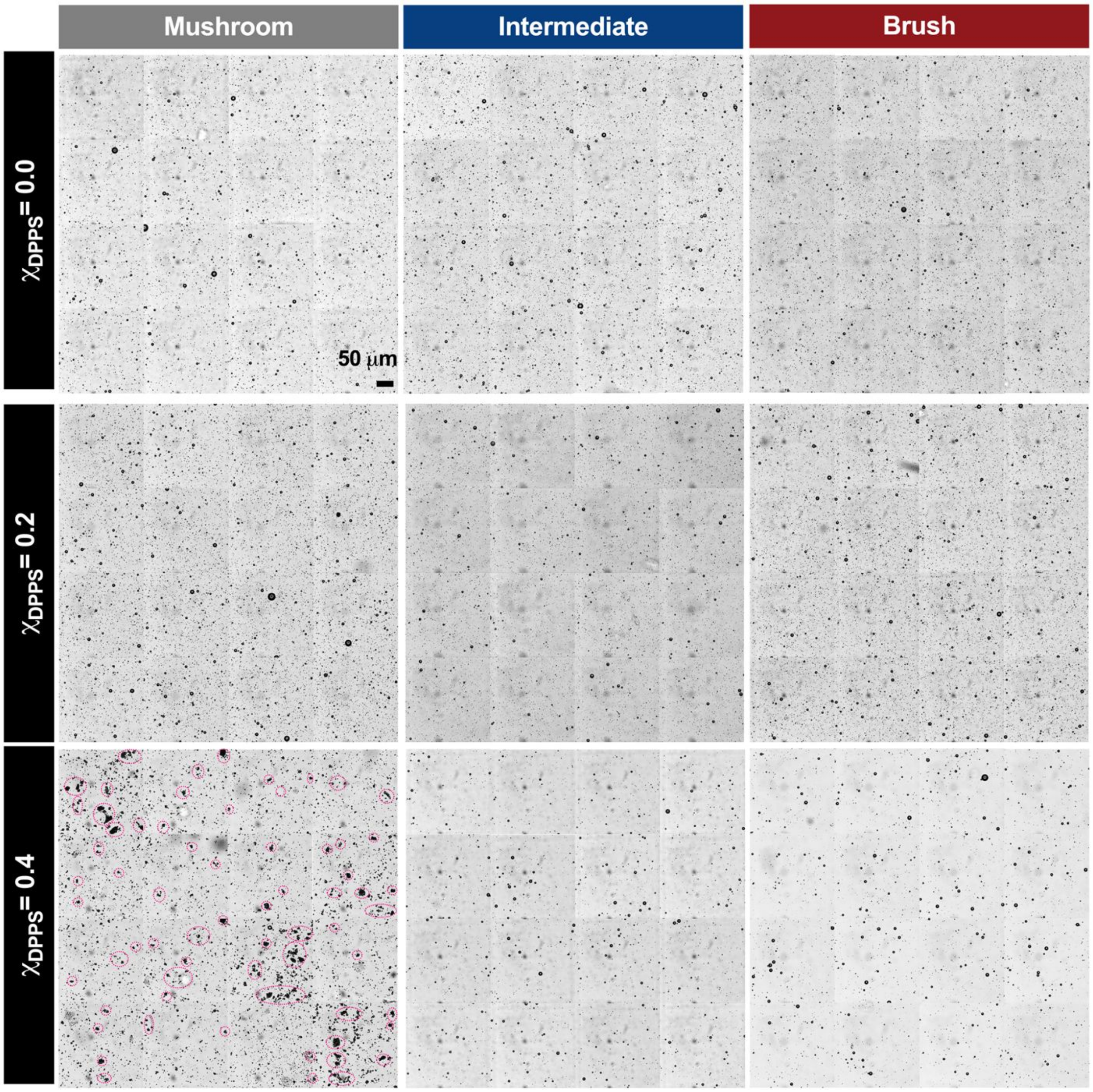

Figure S1. Representative optical microscopy images corresponding to one complete counting quadrant of the Neubauer chamber for DOX−MBs containing different DPPS fractions (χ_{DPPS} = 0.0, 0.2, and 0.4) and exhibiting mushroom, intermediate, and brush PEG architectures. Aggregates are outlined by fuchsia dashed regions. Samples were diluted 1:100 prior to analysis. Each grid square represents an area of 250×250 μm, and the complete counting quadrant measures 1×1 mm. The images were used to characterize PEGylated anionic MB suspensions.

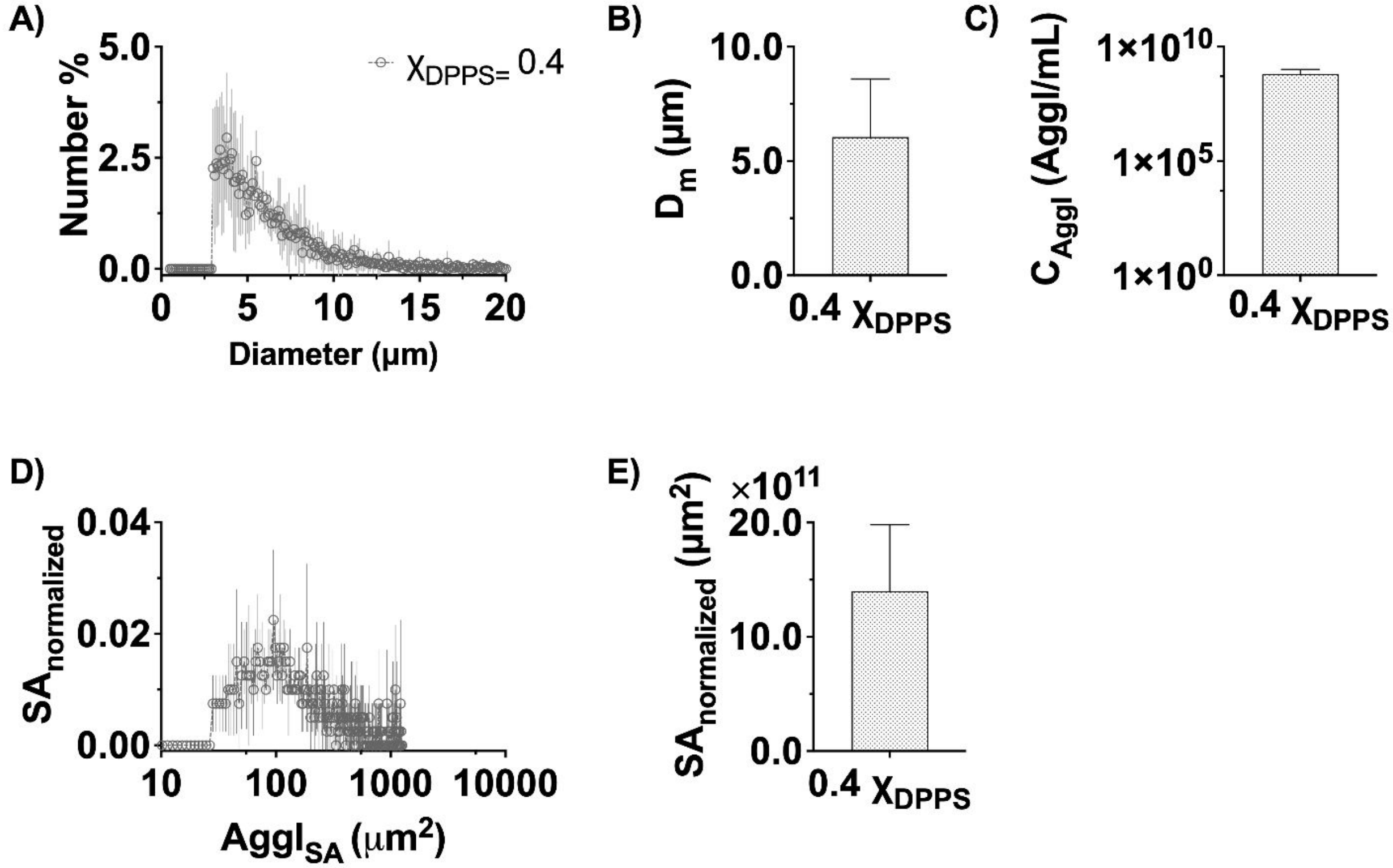

Figure S2. Characterization of agglomerates formed in DOX−MBs exhibiting the mushroom PEG regime at high χ_{DPPS}. A) Number-weighted size distribution, corresponding B) mean agglomerate diameter (D_m), C) agglomerate concentration (C_{Aggl}), and D) surface area-weighted distribution ($SA_{normalized}$), and E) total agglomerate surface area normalized to 1×10^{10} MBs/mL ($SA_{normalized}$). Dashed lines connect adjacent data points. Data represent the mean ± standard deviation of five independent experiments.

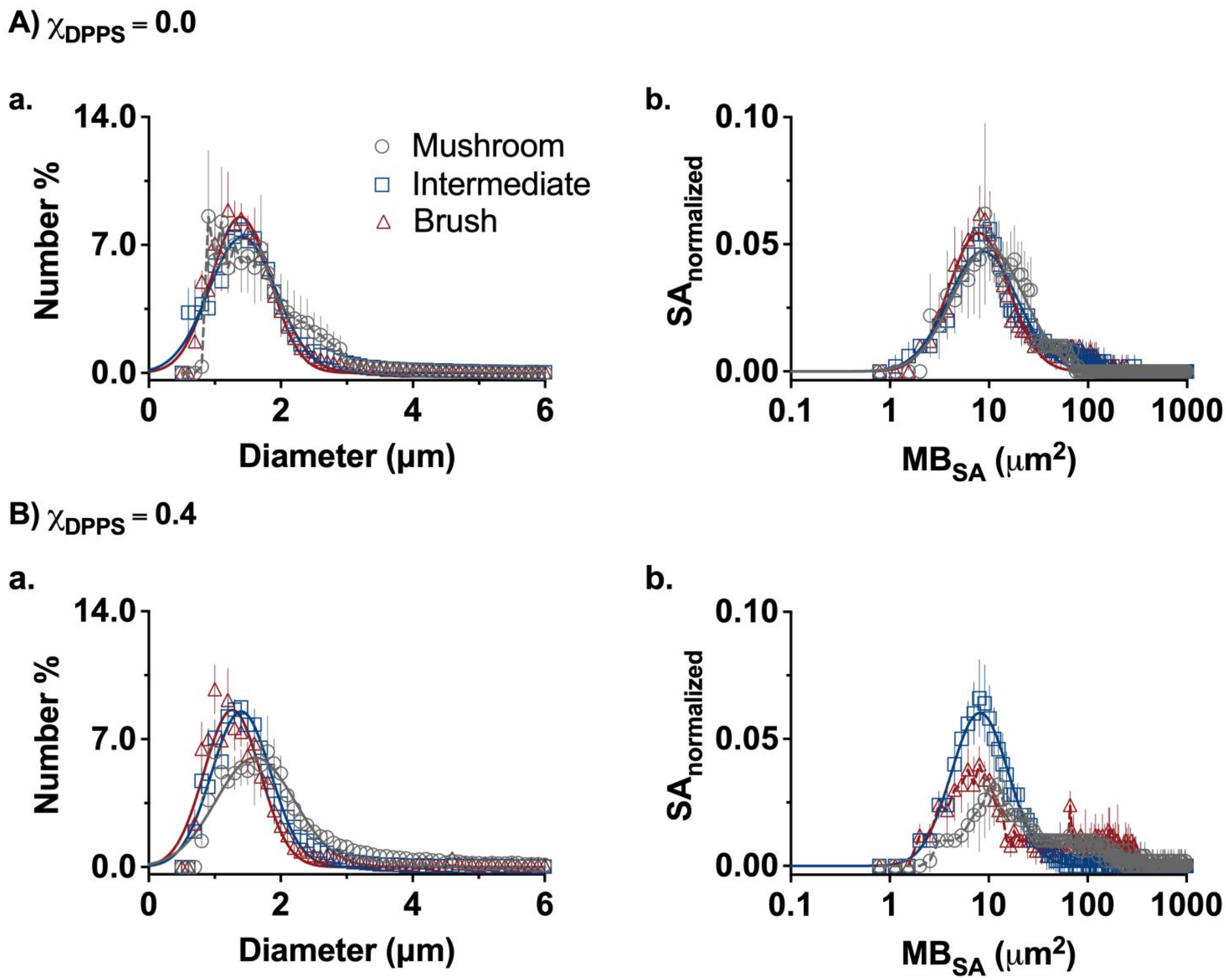

Figure S3. Size and surface area distributions of DOX−MBs with different PEG architectures and χ_{DPPS}. A) χ_{DPPS} = 0.0 and B) χ_{DPPS} = 0.4. (a) Number-weighted size distributions and (b) corresponding total surface area ($SA_{normalized}$) as a function of SA (MB_{SA}), normalized to 1×10^{10} MBs/mL. Solid lines correspond to Gaussian fits ($R^2 \geq 0.95$), and dashed lines connect adjacent data points. Data represent the mean ± standard deviation of five independent experiments.

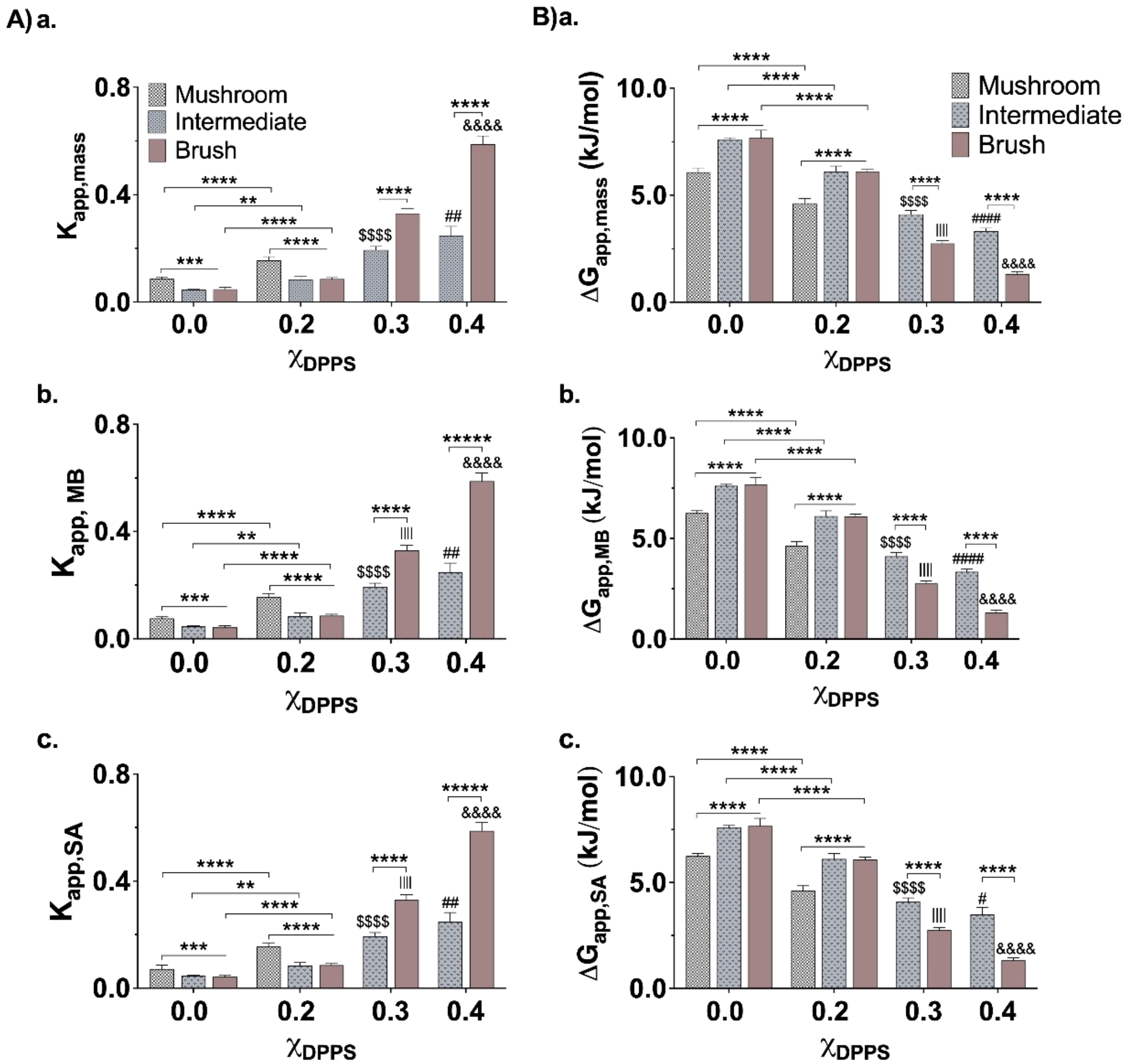

Figure S4. Multiscale apparent adsorption constants and apparent Gibbs free energies of adsorption for DOX−MBs. A) Apparent adsorption constants (K_{app}) obtained from the adsorption trends shown in Figure 2B−D for mushroom, intermediate, and brush PEG architectures as a function of χ_{DPPS}. (a) $K_{app,mass}$, (b) $K_{app,MB}$ and (c) $K_{app,SA}$. (B) Corresponding apparent Gibbs free energies of adsorption (ΔG_{app}) calculated from the corresponding K_{app}. (a) $\Delta G_{app,mass}$, $\Delta G_{app,MB}$, and $\Delta G_{app,SA}$. Data represent the mean ± standard deviation of five independent experiments. Statistical significance is indicated as $^{**,***,****}p<0.05$; $^{\$\$\$\$}p<0.0005$ *vs.* intermediate at $\chi_{DPPS}=0.2$, $^{\#,\#\#,\#\#\#}p<0.05$ *vs.* intermediate at $\chi_{DPPS}=0.3$, $^{||||}p<0.005$ *vs.* brush at $\chi_{DPPS}=0.2$, and $^{\&\&\&\&}p<0.0005$ *vs.* brush at $\chi_{DPPS}=0.3$.

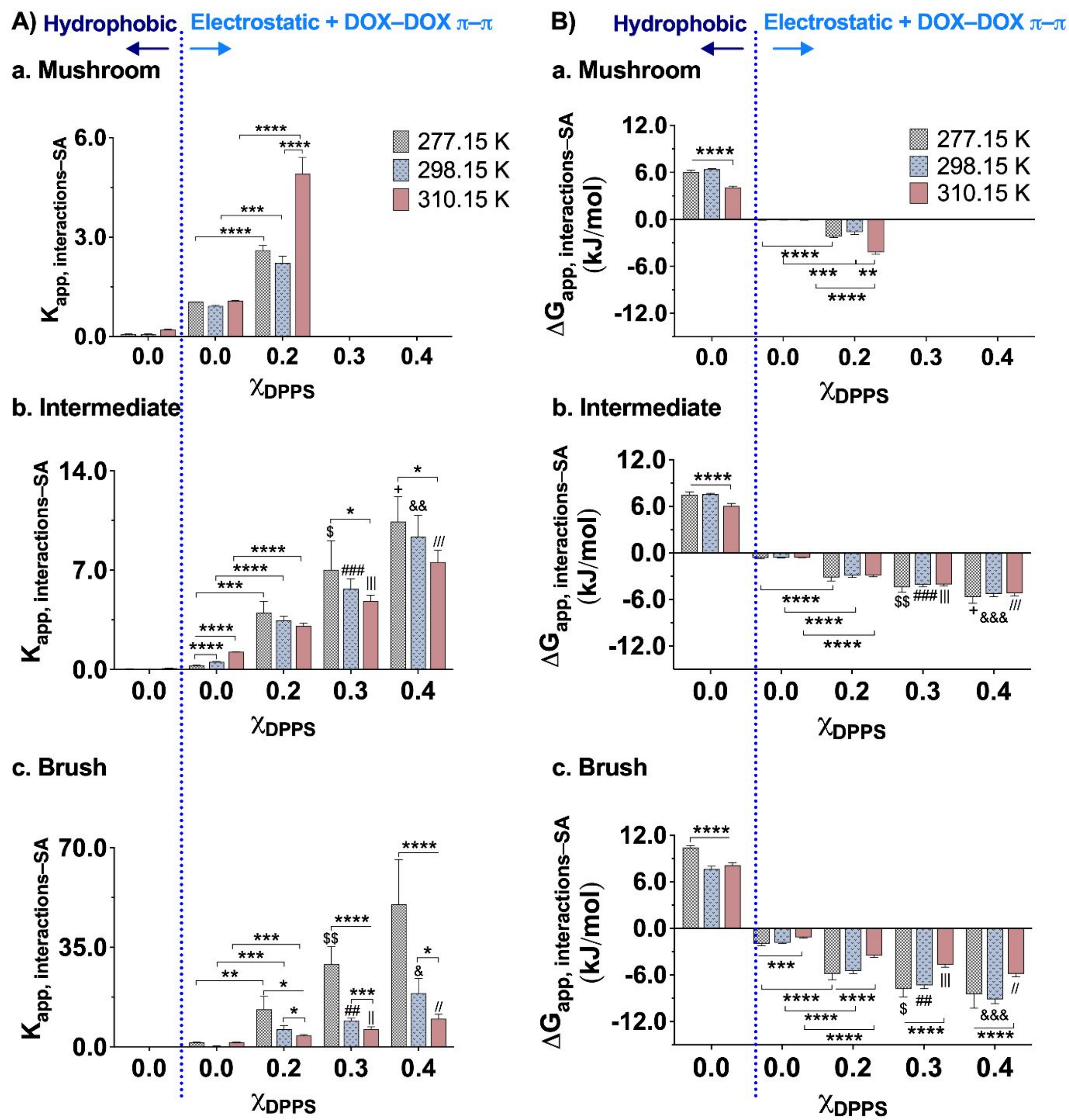

Figure S5. Temperature-dependent thermodynamic analysis of DOX adsorption contributions. A) Apparent adsorption constants ($K_{app,interactions-SA}$) for the hydrophobic and electrostatic + DOX–DOX π–π contributions to DOX adsorption, derived from the surface-area adsorption trends shown in Figure 4A (DOX molecules/μm^2, normalized to 1×10^{10} MBs/mL). Results are shown at 277.15, 298.15, and 310.15 K for the (a) mushroom, (b) intermediate, and (c) brush PEG architectures. B)

Corresponding apparent Gibbs free energy contributions ($\Delta G_{app,interactions-SA}$). MBs were incubated with 0.5 mg/mL DOX for 10 min. Data represent the mean ± standard deviation of five independent experiments. Statistical significance is indicated as $^{*,\ **,\ ***\ ****}p<0.05$; $^{\$,\ \$\$}p<0.05$ *vs.* 277.15 K, $^{\#\#,\#\#\#}p<0.05$ *vs.* 298.15 K, and, $^{\|,\|\|}p<0.05$ *vs.* 310.15 K at $\chi_{DPPS}=0.2$; $^{+}p<0.05$*vs.* 277.15 K, $^{\&,\&\&,\&\&\&}p<0.05$ *vs.* 298.15 K, and,$^{//,///}p<0.05$ *vs.* 310.15 K at $\chi_{DPPS}=0.3$.

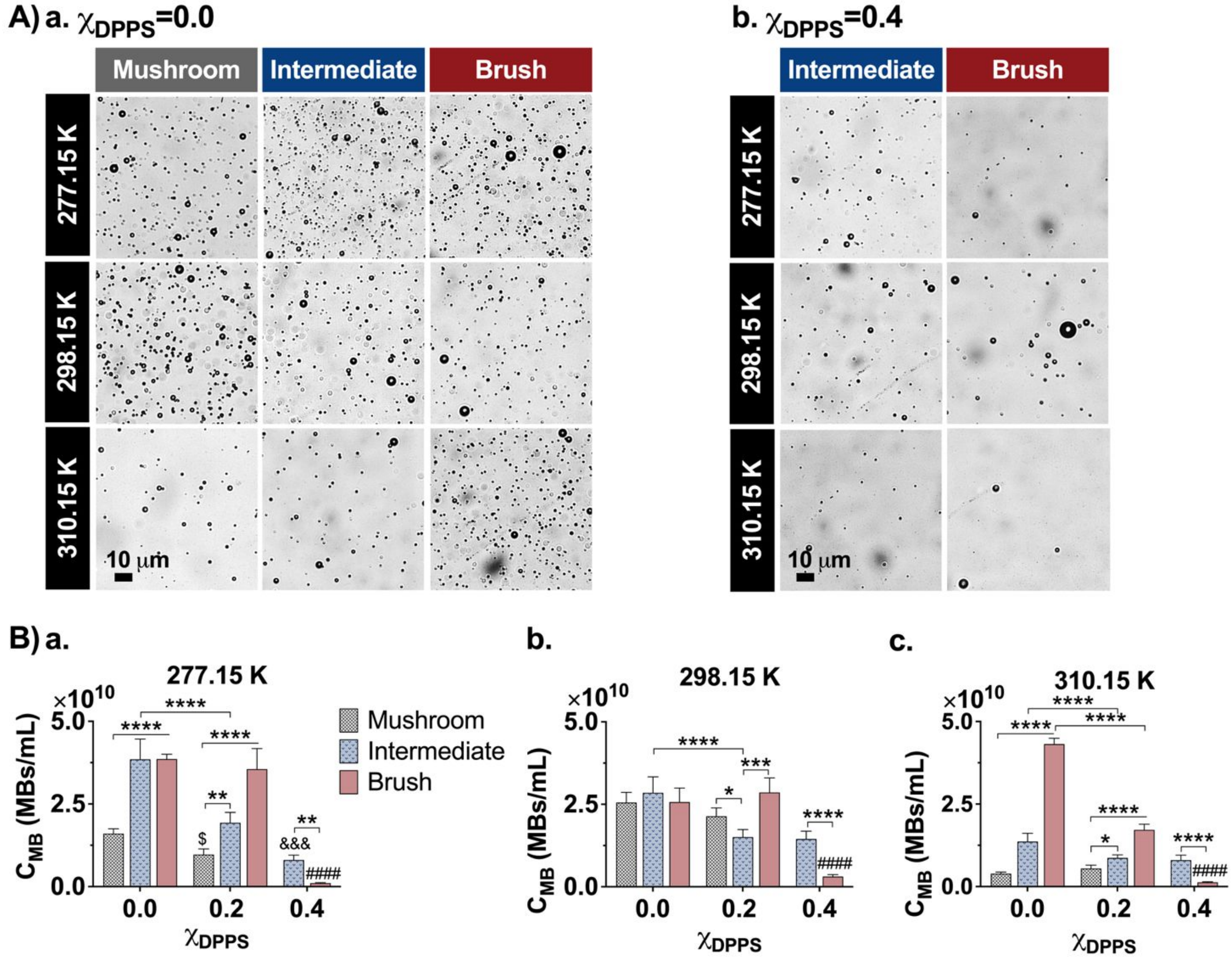

Figure S6. Temperature-dependent variations in DOX–MB concentration across PEG architectures. A) Representative optical microscopy images of DOX–MBs with (a) χ_{DPPS}=0.0 and (b) χ_{DPPS}=0.4 incubated with DOX (0.5 mg/mL, 10 min) at 277.15, 298.15, and 310.15 K. B) DOX–MB concentration (C_{MB}) as a function of χ_{DPPS} at (a) 277.15, (b) 298.15, and (c) 310.15 K. Data represent the mean ± standard deviation of five independent experiments. Statistical significance is indicated as $^{*,\ **,\ ***,\ ****}p<0.05$; $^{\$}p<0.05$ *vs.* mushroom at χ_{DPPS}=0.0; $^{\&\&\&}p<0.005$ *vs.* intermediate at χ_{DPPS}=0.3; $^{\#\#\#\#}p<0.0005$ *vs.* brush at χ_{DPPS}=0.3.

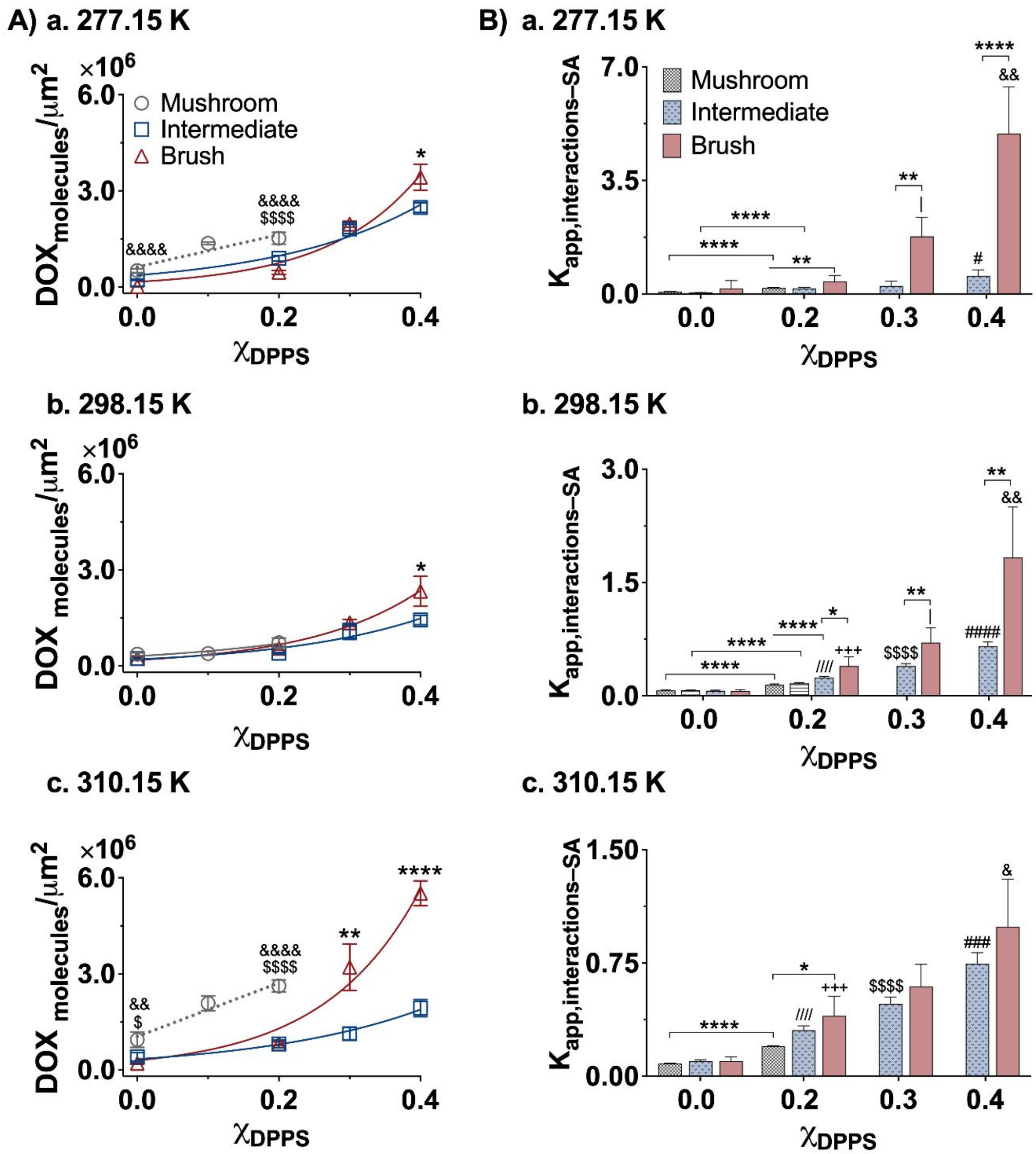

Figure S7. A) Microscopic DOX loading as a function of χ_{DPPS} for mushroom, intermediate and brush PEG architectures at (a) 277.15, (b) 298.15 K, and (c) 310.15 K. Data were normalized to 1×10^{10} MBs/mL. Dotted and solid lines represent linear (R^2>0.95) and exponential (R^2>0.90) adsorption fits, respectively. Statistical comparisons at equivalent χ_{DPPS} are indicated as

$^{\$\$\$\$}$p<0.0005 (mushroom *vs.* intermediate), $^{\&\&, \&\&\&\&}$p<0.05 (mushroom *vs.* brush), and *,**,****p<0.05 (brush *vs.* intermediate). B) Apparent adsorption constants ($K_{app,interactions-SA}$) defined as $K_{app,hydrophobic} + K_{app,electrostatic} + \text{DOX–DOX}_{(\pi\text{-}\pi)}$ at (a) 277.15, (b) 298.15 K, and (c) 310.15 K. MBs were incubated with 0.5 mg/mL DOX for 10 min. Data represent the mean ± standard deviation of five independent experiments. Statistical significance is indicated as $^{////}$p<0.0005 *vs.* intermediate at χ_{DPPS} =0.0, $^{\$\$\$\$}$p<0.05 *vs.* intermediate at χ_{DPPS} =0.2, $^{\#\#\#, \#\#\#\#}$p<0.005 *vs.* intermediate at χ_{DPPS} =0.3, $^{+++}$p<0.0005 *vs.* brush at χ_{DPPS} =0.0, and $^{\&,\&\&}$p<0.05 *vs.* brush at χ_{DPPS} =0.3.

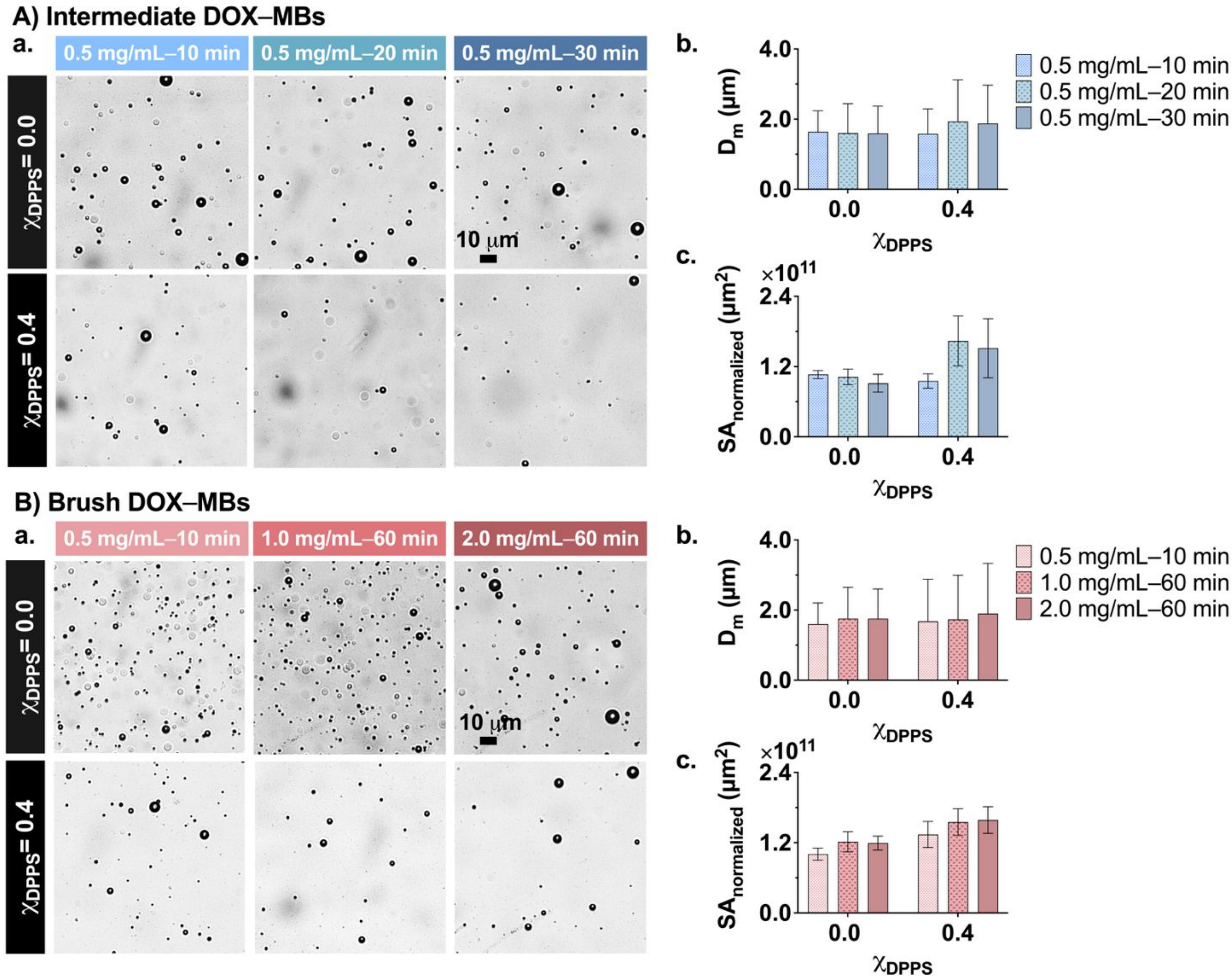

Figure S8. Characterization of intermediate and brush DOX–MB as a function of DOX concentration and incubation time. A) Intermediate DOX–MB and B) brush DOX–MB with χ_{DPPS} = 0.0 and 0.4. MBs were incubated with different DOX concentrations (0.5–2.0 mg/mL) for 10–60 min at 298.15 K. (a) Representative optical microscopy images, (b) mean MB diameter (D_m), and (c) total surface area ($SA_{normalized}$, normalized to 1×10^{10} MBs/mL). Data represent the mean ± standard deviation of five independent experiments.

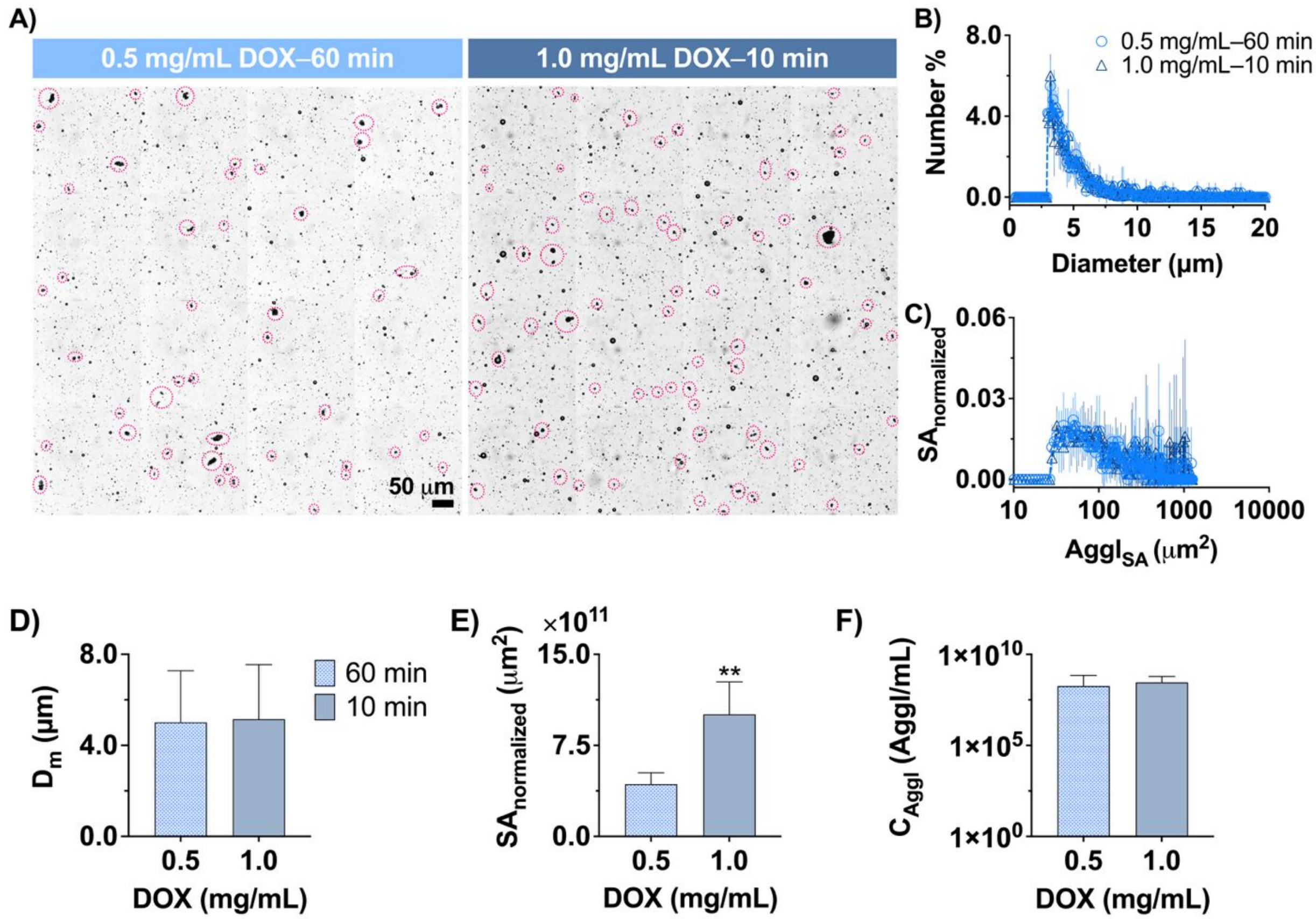

Figure S9. Characterization of DOX−MB agglomerate formation in the intermediate PEG architecture at $\chi_{DPPS} = 0.4$. A) Reconstructed Neubauer chamber quadrant showing 1:100 dilution of intermediate DOX-MBs incubated with 0.5 mg/mL DOX for 60 min and 1.0 mg/mL DOX for 10 min. Agglomerates (Aggl) are outlined by fuchsia dashed regions. B) Number-weighted and C) surface-area size distributions normalized to 1×10^{10} MBs/ mL, D) Aggl diameter (D_m), E) total Aggl surface area ($SA_{normalized}$), and F) Aggl concentration (C_{Aggl}). Data represent the mean ± standard deviation of five independent experiments. Statistical significance is indicated as $^{**}p<0.05$.

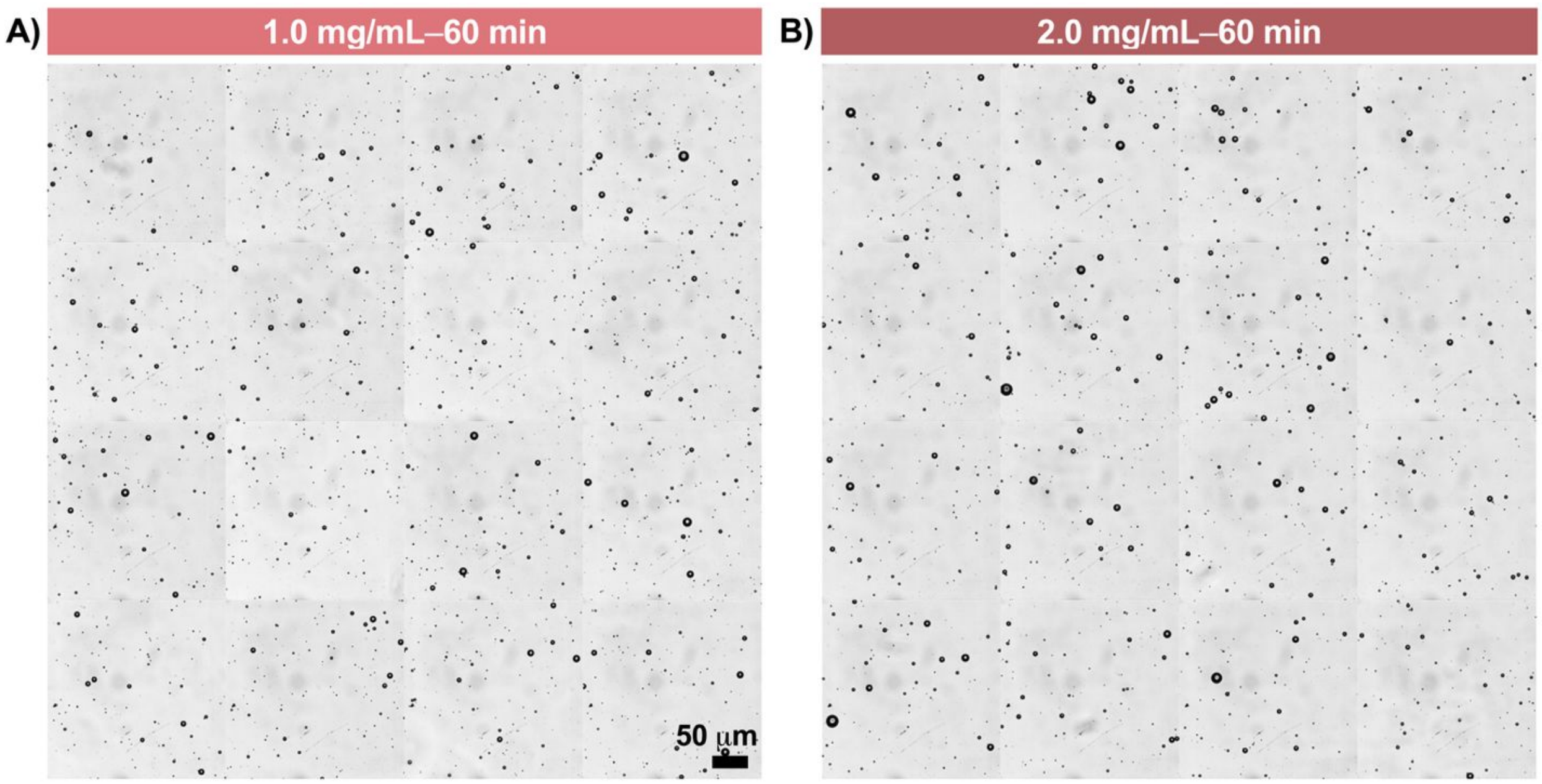

Figure S10. Representative Neubauer chamber reconstructions of DOX-MBs in the brush PEG architecture at $\chi_{DPPS} = 0.4$. A) 1.0 mg/mL DOX and B) 2.0 mg/mL DOX after 60 min incubation. Images correspond to a 1:100 dilution prior to counting.
